# Manganese activates the CBASS immunity to protect bacteria from phage infection

**DOI:** 10.1128/mbio.02758-25

**Published:** 2025-12-08

**Authors:** Xiao Wang, Yongdong Li, Xiao Wang, Wenjing Zhang, Muohua Liu, Xinwei Hao, Shukun Chen, Tianyuan Chang, Conghui Wu, Chonghua Hao, Li Song, Hongxia Ni, Yi Chen, Xihui Shen, Lei Xu

**Affiliations:** 1 State Key Laboratory for Crop Stress Resistance and High-Efficiency Production, Shaanxi Key Laboratory of Agricultural and Environmental Microbiology, College of Life Sciences, Northwest A&F University 12469, Yangling, Shaanxi, China; 2 Ningbo Municipal Center for Disease Control and Prevention 117934, Ningbo, China; 3 Shanxi Provincial People’s Hospital Affiliated to Shanxi Medical University 74648, Taiyuan, China; 4 State Key Laboratory for Crop Stress Resistance and High-Efficiency Production, Shaanxi Key Laboratory of Agricultural and Environmental Microbiology, College of Natural Resources and Environment, Northwest A&F University 12469, Yangling, Shaanxi, China; Harvard Medical School, Boston, Massachusetts, USA

**Keywords:** CBASS, DncV, CapV, manganese, anti-phage

## Abstract

The cyclic-oligonucleotide-based antiphage signaling system (CBASS) is essential for bacterial defense against phage infections, mirroring many features of the eukaryotic cGAS-STING pathway. Although metal ions are well-known regulators of cGAS-STING activation, their impact on CBASS-mediated antiviral function remains largely unexplored. Here, we show that manganese (Mn^2+^) serves as a key cofactor to enhance CBASS activation. Upon phage infection, the intracellular Mn^2+^ level is elevated, and the gene expression of the Mn^2+^ transportation system is upregulated. We found that Mn^2+^ directly augments the activity of DncV, markedly boosting 3′3′-cGAMP production. Consequently, the phospholipase CapV is activated more rapidly, driving premature bacterial cell lysis and curtailing phage replication. Notably, Mn^2+^ also alleviates folate-mediated inhibition of DncV, underscoring its role as a potent modulator of cyclic dinucleotide signaling. Our findings reveal a mechanism through which Mn^2+^ confers bacterial resistance to phages, mirroring the Mn^2+^-enhanced antiviral responses of mammalian cGAS-STING.

**IMPORTANCE:**

Bacteriophages pose a persistent threat to bacterial survival, driving the evolution of diverse antiviral systems, including the cyclic-oligonucleotide-based antiphage signaling system (CBASS) immunity. Here, we reveal that manganese (Mn^2+^) acts as a pivotal cofactor for CBASS, directly enhancing the activity of the cGAS-like cyclase DncV to generate 3′3′-cGAMP, which, in turn, activates the phospholipase CapV. This Mn^2+^-driven DncV activation induces rapid bacterial cell death, thereby limiting phage replication. These findings underscore a striking parallel with mammalian cGAS-STING, where Mn^2+^ likewise amplifies antiviral responses. By illuminating the importance of Mn^2+^ homeostasis in bacterial phage resistance, our study broadens the understanding of bacterial innate immunity and highlights a deeply conserved mechanism across prokaryotes and eukaryotes.

## INTRODUCTION

Bacteriophages (phages) rank among the most abundant biological entities on Earth and pose a constant threat to bacterial survival. Over billions of years of co-evolution, bacteria have evolved numerous strategies to detect, counteract, and limit phage replication. Among these strategies, the cyclic-oligonucleotide-based antiphage signaling system (CBASS) has gained significant attention as a newly recognized and potent antiviral mechanism (1). Unlike other well-characterized defense systems, such as Restriction-Modification (RM) and Clustered Regularly Interspaced Short Palindromic Repeats and CRISPR-associated proteins (CRISPR-Cas) system (2), which directly target phage genetic material by cleaving invading DNA, CBASS employs a more indirect approach (2–4). CBASS functions by generating cyclic nucleotide signals like 3′3′-cGAMP upon phage invasion, leading to the activation of effector proteins, such as the phospholipase CapV that induce bacterial cell death before the phage completes its replication cycle (5). Once the phage is detected, a specialized nucleotidyl cyclase synthesizes specific cyclic oligonucleotide molecules. These molecules, often referred to as second messengers, activate a downstream effector, commonly a phospholipase, nuclease, or pore-forming protein, triggering cell death that causes abortive infection (6). This abortive process is conceptually similar to eukaryotic innate immunity, where infected cells undergo apoptosis to confine viral spread (7). Numerous genomic surveys have revealed thousands of distinct CBASS variants across bacterial and archaeal genomes, underscoring their prevalence and evolutionary significance (1, 3, 8).

One of the most interesting aspects of CBASS is its unexpected similarity to the eukaryotic cGAS-STING pathway (7, 9, 10). In mammalian cells, the enzyme cyclic GMP-AMP synthase (cGAS) detects cytosolic double-stranded DNA (dsDNA), often indicative of viral infection or cellular damage, and catalyzes the formation of 2′3′-cGAMP from ATP and GTP (11). The 2′3′-cGAMP binds to and activates STING (stimulator of interferon genes), which then triggers a cascade involving kinases such as TBK1, culminating in the phosphorylation and nuclear translocation of transcription factors like IRF3 that promote interferon and other cytokine gene expression (12). The cGAS-STING forms a keystone of innate immunity in vertebrates, driving robust antiviral and antitumor responses. Surprisingly, homologous nucleotidyl cyclases, which are structurally reminiscent of cGAS, have been characterized in diverse bacterial species. The enzyme DncV in *Vibrio cholerae*, for example, displays a cGAS-like fold and produces 3′3′-cGAMP, an isomer different from mammalian 2′3′-cGAMP but with a shared function as a second messenger (9). Such bacterial cyclases, paired with effector proteins (e.g., the phospholipase CapV), form the core of the CBASS system (1). This resemblance extends beyond mere structural homology: both cGAS and bacterial cGAS-like enzymes rely on producing cyclic oligonucleotides to activate effector pathways that can lead to protective outcomes. These parallels suggest that the fundamental concept of cyclic nucleotide-mediated immunity may have ancient evolutionary roots. Indeed, mounting evidence points to a scenario in which the ability of STING to sense cyclic dinucleotides (CDNs) originally arose in prokaryotes, later co-opted and refined by eukaryotes for a broader antiviral function (13, 14).

The role of manganese (Mn^2+^) in enhancing host innate immune response has been reported recently, particularly the cGAS-STING pathway signaling (12, 15–19). Mammalian cGAS relies on divalent cations (usually Mg^2+^) in its catalytic center to properly align ATP and GTP for cyclization. However, compelling data show that Mn^2+^ can magnify cGAS activity by orders of magnitude (19). Even low concentrations of Mn^2+^ can drastically lower the threshold for dsDNA detection, accelerate 2′3′-cGAMP synthesis, and render the cGAS-STING pathway hyper-responsive (19, 20). This activation also extends to STING that Mn^2+^ can bolster STING’s affinity for cGAMP, improving signal propagation downstream (18, 19). Beyond purely antiviral roles, Mn^2+^-augmented STING signaling has also drawn attention in the antitumor arena (12, 16). Given the structural and functional similarities between bacterial cGAS-like enzymes and mammalian cGAS (9), it is plausible that Mn^2+^ exerts a similarly enhancing effect on bacterial cyclic nucleotide synthases. However, while the immunomodulatory properties of Mn^2+^ in mammalian cells are well-documented, its role in bacterial antiviral defenses remains largely unexplored.

Mn^2+^ is an essential trace element for bacteria (21). It participates extensively in critical cellular processes such as energy metabolism, DNA biosynthesis, and transcriptional regulation, while playing a central role in oxidative stress responses (22, 23). In bacterial cells, physiological intracellular Mn^2+^ concentrations generally range from 10 to 100 μM under basal conditions, depending on species and environmental availability (e.g., ~20–50 µM in *Escherichia coli* during exponential growth) (21, 24). Extracellular Mn^2+^ in natural environments (e.g., soil or host tissues) is often lower (~1–10 µM) (25), creating a gradient that necessitates active uptake for homeostasis. During stress, such as oxidative bursts or metal scarcity, bacteria can rapidly elevate intracellular Mn^2+^ to millimolar levels. This Mn^2+^ serves as an essential cofactor for reactive oxygen species (ROS)-detoxifying enzymes such as superoxide dismutase (SodA) and catalase (KatN), assembling functional antioxidant complexes while suppressing hydroxyl radical accumulation by inhibiting the Fenton reaction, thereby delivering dual antioxidant protection. Under oxidative stress, the Fenton reaction generates cytotoxic hydroxyl radicals that threaten bacterial survival; Mn^2+^ mitigates this toxicity by reducing intracellular radical levels, thereby enhancing bacterial viability (26, 27). Simultaneously, under metal-scarce conditions, bacteria raise their intracellular Mn^2+^ reserve to guarantee the activity of key Mn^2+^-dependent enzymes. To cope with environmental stresses, bacteria have evolved a diverse array of Mn^2+^ transporters, including the SitABCD, ABC-type permeases, the Nramp family transporter MntH, and T6SS-mediated Mn^2+^ acquisition systems (18, 24, 26, 28, 29). Critically, the dynamic regulation of these transporters enables bacteria to rapidly rewire Mn^2+^ homeostasis in response to environmental fluctuations. The universal requirement of Mn^2+^ for numerous bacterial metabolic enzymes, such as manganese-dependent SodA (21, 30), implies that during infection, bacteria must carefully regulate Mn^2+^ homeostasis. Bacteria like *Salmonella enterica serovar* Typhimurium and *E. coli* often upregulate Mn^2+^ import systems to sustain virulence and survive in hostile, metal-scarce environments (8, 27). However, no studies showed that bacterial cells can also exploit these transporters to stockpile Mn^2+^ to activate anti-phage defenses. Thus, the potential significance of bacterial metal-ion regulation in the context of phage infection needs further investigation.

In this study, we investigated how Mn^2+^ might influence the anti-phage efficacy of the CBASS system from the *V. cholerae* El Tor N16961 strain. We showed that intracellular Mn^2+^ was accumulated upon phage infection. We further demonstrated that Mn^2+^ amplifies the DncV reaction rate, leading to accelerating cGAMP synthesis and bypassing the inhibitory effects of folate. Although Mn^2+^ does not directly stimulate CapV phospholipase activity, the elevated production of 3′3′-cGAMP under Mn^2+^ conditions leads to enhanced CapV activity. Moreover, bacterial strains with increased intracellular Mn^2+^ exhibit enhanced survival and reduced phage yield, while genetic disruption of Mn^2+^ homeostasis alters the anti-phage ability of CBASS, highlighting the crucial role of metal-ion regulation in antiviral immunity. In sum, our findings reveal that Mn^2+^ functions as a key cofactor for bacterial cGAS-like cyclases. This phenomenon mirrors the Mn^2+^-augmented cGAS-STING axis in mammalian immunity, reinforcing the notion that fundamental themes in innate immunity, the synergy between metal-ion cofactors and nucleotidyltransferase sensors, can be strikingly conserved across prokaryotes and eukaryotes.

## RESULTS

### Phage infection leads to the accumulation of Mn^2+^ in bacteria

**Fig 1:**
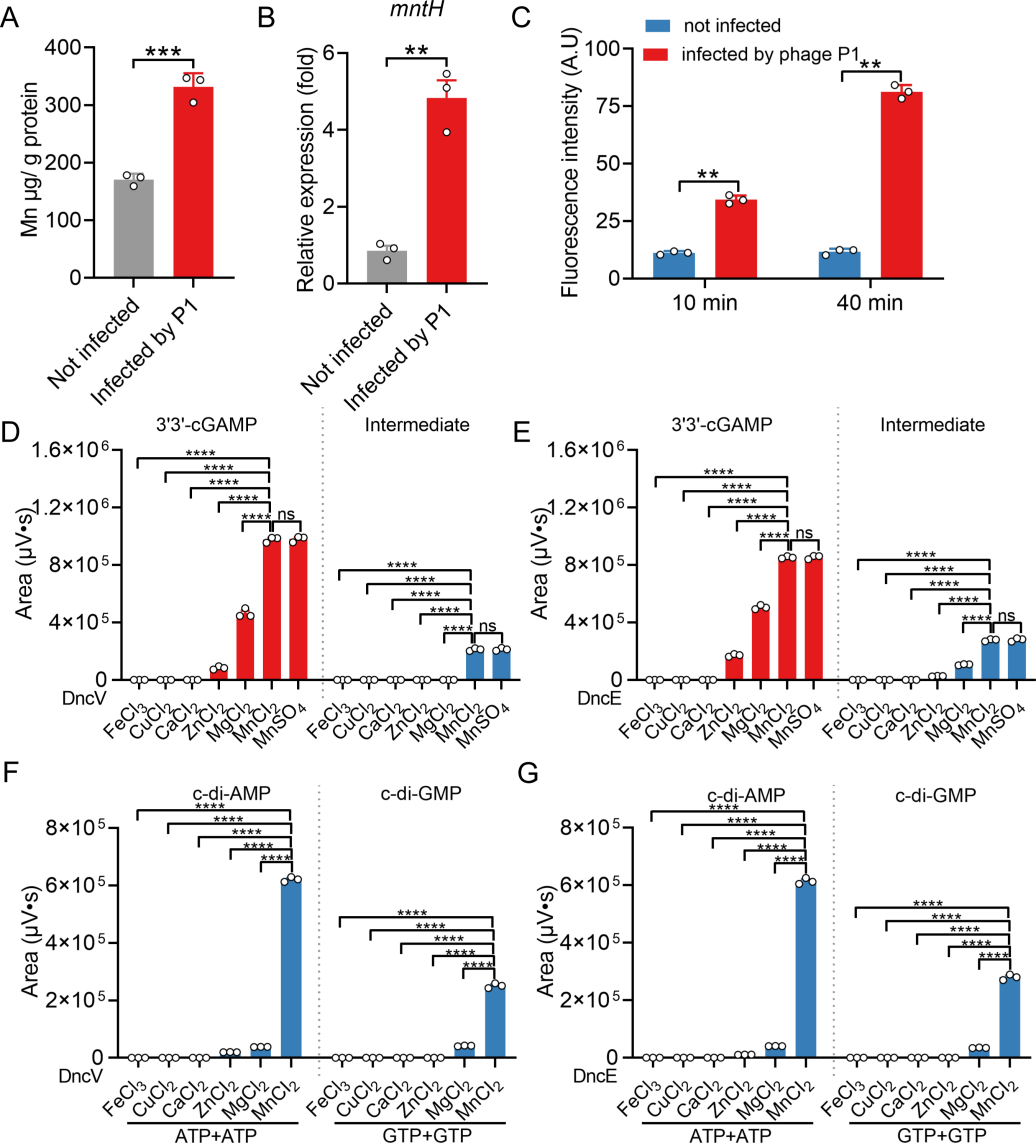
The phage infection increases intracellular Mn^2+^ concentrations, and Mn^2+^ enhances the ability of DncV to synthesize CDNs. (**A**) P1 phage-infected bacteria K12-MG1655 for 10 min (multiplicity of infection [MOI] = 0.1), the infected bacteria were centrifuged at 4,500 rpm for 20 min to collect bacterial precipitation and washed with PBS buffer, and Mn^2+^ associated with the bacterial cell was measured by inductively coupled plasmon resonance atomic absorption spectrometry (ICP-MS). Uninfected bacteria were used as the control group. (**B**) P1-infected K12-MG1655 for 10 min (MOI = 0.1), then the bacteria were collected and total RNA extracted. The expression of *mntH* was analyzed by qRT-PCR and normalized to *16S*. (**C**) P1 phage-infected bacteria for 10 or 40 min (MOI = 0.1), the infected bacteria were centrifuged at 4,500 rpm for 25 min to collect bacterial precipitation and washed with PBS buffer. The intracellular levels of ROS were determined with H_2_DCFDA dye. H₂DCFDA fluorescence was measured at 488/525 nm (ex/em) using a SpectraMax M2 plate reader. (**D**) and (**E**) 2 µM protein DncV (**D**) or DncE (**E**) was added into the reaction system (50 mM Tris-HCl, pH 7.5, 1 mM metal ions, 100 µM ATP, and 100 µM GTP) for 30 min at 37°C. The metal salts include FeCl_3_, CuCl_2_, CaCl_2_, ZnCl_2_, MgCl_2_, MnCl_2_, and MnSO_4_. Next, all reaction mixtures were heated for 10 min at 98°C and centrifuged for 10 min at 12,000 rpm to obtain supernatant. Lastly, all samples were detected by high-performance liquid chromatography (HPLC). The wavelength of the UV detector was 254 nm. The supernatant was eluted with 98% Na_2_HPO_4_ (pH 5.2, 150 mM) and 2% acetonitrile in 30 min, and the flow rate was 1 mL/min. The production levels of 3′3′-cGAMP and intermediate products under varying reaction conditions were quantitatively analyzed using Empower software. (**F**) and (**G**) Using ATP or GTP as the sole substrate, 2 μM protein DncV (**F**) or DncE (**G**) was added into the reaction system (50 mM Tris-HCl, pH 7.5, 1 mM metal ions, 100 µM ATP, or 100 µM GTP) for 30 min at 37°C. The metal salts include FeCl_3_, CuCl_2_, CaCl_2_, ZnCl_2_, MgCl_2_, and MnCl_2_. All other manipulations were as above. The production levels of c-di-AMP or c-di-GMP products under varying reaction conditions were quantitatively analyzed using Empower software. Data represent the mean ± SEM of three biological replicates, each of which was performed with three technical replicates. *P*  values were calculated using a two-tailed Student’s *t*-test for paired comparisons or a one-way analysis of variance (ANOVA) for multiple comparisons. **P < 0.01; ***P < 0.001; ****P < 0.0001; ns, not significant.

In eukaryotic cells, the distribution of transition metals is changed during viral and bacterial infection, and those metal ions play crucial roles in the interplay between the host and the pathogen (18, 19). In particular, Mn^2+^ is essential for the antiviral defense of eukaryotic cells. We investigated whether phage infection in bacterial cells could also affect the distribution of transition metals like Mn^2+^. To assess whether bacteriophage infection influences the intracellular distribution of transition metals in *E. coli*, we measured the levels of several metal ions following phage infection using inductively coupled plasma mass spectrometry (ICP-MS). We focused on manganese (Mn^2+^) as a representative of key transition metals that affect innate antiviral immunity in eukaryotic cells. When *E. coli* K12-MG1655 wild-type cells were infected with phage P1 for 10 min, the Mn^2+^ concentration in the bacterial pellet fraction approximately doubled relative to the control (Fig. 1A), suggesting a marked increase in intracellular Mn^2+^ content compared to non-infected cells. In addition to Mn^2+^, we noted a modest but reproducible elevation in Mg^2+^ levels under P1 infection conditions (Fig. S1). These data indicate that P1 phage infection triggers a response that leads to the accumulation of Mn^2+^ and, to a lesser degree, Mg^2+^ within the bacterial cytoplasm. To understand the molecular basis underpinning Mn^2+^ accumulation, we analyzed the expression of genes involved in Mn^2+^ uptake. The expression of the manganese transportation system was also induced during phage infection, as shown by the upregulation of Mn^2+^ transporter gene *mntH*, suggesting that phage infection prompts the bacterial cell to activate its Mn^2+^ import machinery (Fig. 1B). Moreover, to determine whether Mn^2+^ accumulation is a general feature of phage infection rather than a P1-specific phenomenon, we examined changes in metal levels upon infection with a genetically distinct phage, T4. Similar to the P1 infection scenario, T4-infected cells showed a substantial rise in intracellular Mn^2+^ compared to uninfected controls, with Mn^2+^ levels increasing by several folds (Fig. S2A). Concomitantly, intracellular Mg^2+^ also exhibited a measurable increase under T4 infection (Fig. S2B). Consistently, the expression of *mntH* was induced during T4 phage infection (Fig. S2C). These findings suggest that phage infection commonly drives Mn^2+^ and Mg^2+^ accumulation within bacterial cells, irrespective of the phage type. It is well established that during bacterial antioxidant responses, Mn^2+^ acts as an essential cofactor for ROS-detoxifying enzymes (e.g., superoxide dismutase SodA and catalase KatN), forming Mn^2+^-dependent antioxidant complexes to scavenge ROS and mitigate oxidative stress (22, 23, 31). Further investigations into the downstream effects of such ion accumulation revealed that, regardless of whether it was the P1 phage or the T4 phage, phage infection of bacteria K12-MG1655 led to an increase in the level of ROS within bacterial cells (Fig. 1C ; Fig. S2D).

### Mn^2+^ directly activates the DncV *in vitro*

Divalent ions play essential roles in the activation of cGAS, and Mn^2+^ enhances the activity of cGAS in the production of 2′3′-cGAMP (19). Similar to mammalian cells, the homolog of the gene *dncV* (dinucleotide cyclase in *Vibrio*, VC0179) encoded cGAS was also identified in *V. cholerae* (9, 32). To determine how divalent ions influence the catalytic activity of bacterial dinucleotide cyclases, we first examined the activity of *V. cholerae* DncV, a homolog of mammalian cGAS, in producing 3′3′-cGAMP. High-performance liquid chromatography (HPLC) analysis confirmed that the product generated by DncV eluted with the same retention time as a commercial 3′3′-cGAMP standard, establishing the identity of the reaction product (Fig. S3A). Quantification based on a standard curve further supported the accurate measurement of 3′3′-cGAMP yields (Fig. S3B).

Using ATP and GTP as the substrate pair, we assessed the production of the signaling molecule 3′3′-cGAMP by adding various divalent ions (Fe^3+^, Cu^2+^, Ca^2+^, Zn^2+^, Mg^2+^, and Mn^2+^) as cofactors. Among the tested divalent ions, Mn^2+^ or Mg^2+^ enabled a robust production of 3′3′-cGAMP, whereas Zn^2+^ supported only a lower level of activity. No detectable enzymatic activity was observed with Ca^2+^, Cu^2+^, or Fe^3+^, demonstrating that not all ions can serve as functional cofactors (Fig. 1D). Strikingly, the use of Mn^2+^ (MnCl_2_ or MnSO_4_) as a cofactor resulted in a significantly higher yield of 3′3′-cGAMP than those obtained with Mg^2+^ or Zn^2+^. Moreover, the intermediate products were only detected in the reaction system mediated by Mn^2+^, whether using MnCl_2_ or MnSO_4_ (Fig. 1D). This finding indicates that Mn^2+^ is uniquely effective in enhancing DncV’s catalytic efficiency for producing 3′3′-cGAMP and intermediate.

We next assessed whether this effect of Mn^2+^ is conserved in other bacterial dinucleotide cyclases. To this end, we identified a homolog of *dncV* in *E. coli* (Fig. S4A and B), referred to as DncE (dinucleotide cyclase in *E. coli*). Similar to DncV, DncE also produced 3′3′-cGAMP and intermediate at markedly elevated levels in the presence of Mn^2+^ (MnCl_2_ or MnSO_4_) compared with Mg^2+^ or Zn^2+^ (Fig. 1E). Importantly, Mn^2+^ did not alter the reaction’s product identity, only its efficiency, further highlighting the ion’s ability to enhance 3′3′-cGAMP synthesis without changing the enzyme’s substrate specificity or reaction outcome. This result suggests that the stimulatory effect of Mn^2+^ on 3′3′-cGAMP synthesis is conserved between at least two distinct bacterial dinucleotide cyclase enzymes (Fig. 1D and E). With sequence alignment, we found DncV is highly conserved in bacteria, and this residue was also conserved in several other cGAS-like proteins (Fig. S4C).

We further explored whether Mn^2+^ influences the substrate specificity of DncV. This enzyme can also form CDNs, such as c-di-AMP and c-di-GMP, when provided with single-substrate pairs (ATP/ATP or GTP/GTP), respectively (32). In the presence of Mg^2+^, only trace amounts of c-di-AMP or c-di-GMP were detected. Strikingly, when Mn^2+^ was used as the cofactor, DncV or DncE generated significantly higher levels of both c-di-AMP and c-di-GMP, indicating that Mn^2+^ not only enhances activity with the cognate substrate pair but also expands the effective substrate utilization (Fig. 1F and G). Under these conditions, reactions containing Fe^3+^, Cu^2+^, Ca^2+^, or Zn^2+^ again produced negligible amounts of these alternative CDN, underscoring the unique and robust activating role of Mn^2+^ (Fig. 1F and G). These findings highlight Mn^2+^ as a key cofactor that promotes the production of bacterial CDNs, paralleling the reported role of Mn^2+^ in enhancing cGAS activity in mammalian cells (19).

### Mn^2+^ activates DncV dependent on the key enzymatic sites and independent of the ligand

**Fig 2:**
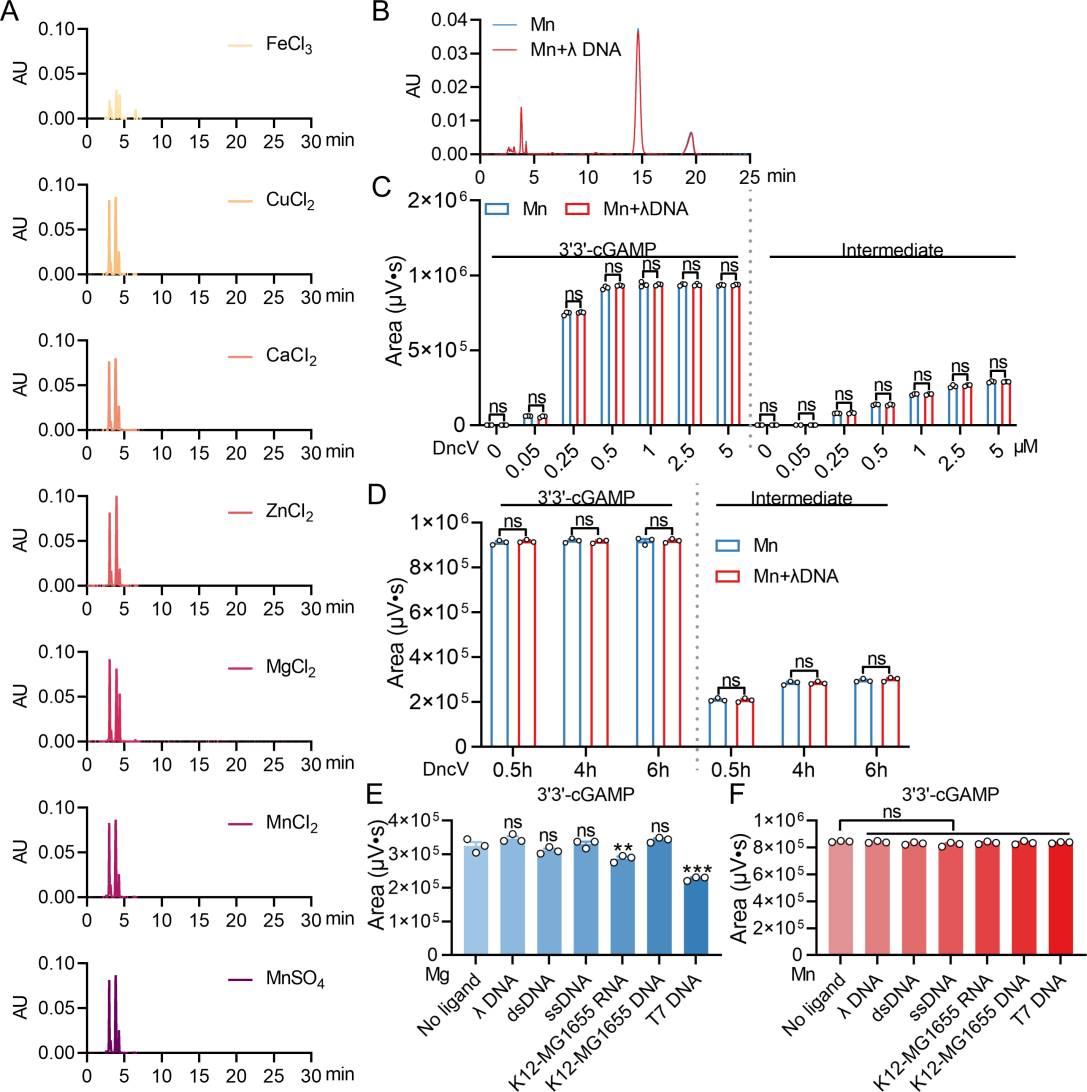
Mn^2+^ enhances the ability of DncV to synthesize 3'3'-cGAMP, dependent on the key enzymatic sites but independent of ligand. (**A**) 2 µM double-point mutant protein DncV^D131A/D133A^ was added into the reaction system (50 mM Tris-HCl, pH 7.5, 1 mM metal ions, 100 µM ATP, and 100 µM GTP) for 30 min at 37°C. The metal salts include FeCl_3_, CuCl_2_, CaCl_2_, ZnCl_2_, MgCl_2_, MnCl_2_, and MnSO_4_. Next, all reaction mixtures were heated for 10 min at 98°C and centrifuged for 10 min at 12,000 rpm to obtain supernatant. Lastly, all samples were detected by HPLC. (**B**) In the presence or absence of ligand λ DNA, 2 µM protein DncV was added into the reaction system (50 mM Tris-HCl, pH 7.5, 1 mM MnCl_2_, 100 µM ATP, and 100 µM GTP) for 30 min at 37°C. Next, all reaction mixtures were heated for 10 min at 98°C and centrifuged for 10 min at 12,000 rpm to obtain supernatant. Lastly, all samples were detected by HPLC. (**C**) In the presence or absence of ligand λ DNA, 0, 0.05, 0.25, 0.5, 1, 2.5, or 5 µM protein DncV was added into the reaction system (50 mM Tris-HCl, pH 7.5, 1 mM MnCl_2_, 100 µM ATP, and 100 µM GTP) for 30 min at 37°C, respectively. All reaction mixtures were heated for 10 min at 98°C and centrifuged for 10 min at 12,000 rpm to obtain supernatant. Lastly, all samples were detected by HPLC. The production levels of 3'3'-cGAMP and intermediate products in the Mn^2+^-mediated or Mn^2+^-λ DNA-mediated reaction system were quantitatively analyzed using Empower software. (**D**) In the presence or absence of ligand λ DNA, 2 µM protein DncV was added into the reaction system (50 mM Tris-HCl, pH 7.5, 1 mM MnCl_2_, 100 µM ATP, and 100 µM GTP) for 0.5 h, 4 h, or 6 h at 37°C. All other manipulations were as above. The production levels of 3'3'-cGAMP and intermediate products in the Mn^2+^-mediated or Mn^2+^-λ DNA-mediated reaction system were quantitatively analyzed using Empower software. (**E**) and (**F**) Under the reaction conditions of no ligand, λ DNA, dsDNA, ssDNA, K12-MG1655 RNA, K12-MG1655 DNA, or T7 DNA in Mg^2+^-mediated (**E**) or Mn^2+^-mediated (**F**) reaction system, 2 µM protein DncV was added into the reaction system (50 mM Tris-HCl, pH 7.5, 1 mM MgCl_2_ or MnCl_2_, 100 µM ATP, and 100 µM GTP) for 30 min at 37°C. Next, all reaction mixtures were heated for 10 min at 98°C and centrifuged for 10 min at 12,000 rpm to obtain supernatant. Lastly, all samples were detected by HPLC. The production levels of 3'3'-cGAMP in the Mg^2+^-mediated or Mn^2+^-mediated reaction system were quantitatively analyzed using Empower software. Data represent the mean ± SEM of three biological replicates, each of which was performed with three technical replicates. *P*  values were calculated using a two-tailed Student’s *t*-test for paired comparisons one-way analysis of variance (ANOVA) for multiple comparisons. ns, not significant.

To investigate the Mn^2+^-dependent activation of DncV and determine whether it is influenced by the enzyme’s known catalytic residues or DNA ligands, we first examined a DncV variant bearing substitutions at its essential catalytic sites. Previous studies identified Asp131 and Asp133 as critical residues for DncV enzymatic activity (32). We purified a D131A/D133A double mutant and tested its activity in the presence of various metal ions. HPLC analysis revealed that, unlike the WT protein, this mutant enzyme produced no detectable products, including both the 3′3′-cGAMP and the intermediates, even when supplemented with Mn^2+^ (Fig. 2A). This finding confirms that the integrity of these key catalytic residues is indispensable for DncV function and that Mn^2+^ enhancement cannot compensate for the loss of these essential active-site residues.

Next, we explored whether DncV activation by Mn^2+^ is influenced by DNA ligands. In mammalian cells, cGAS can be activated via both a canonical dsDNA-dependent pathway and a ligand-independent mechanism mediated by Mn^2+^ (33). However, our *in vitro* experiments found that the catalytic activity of DncV is independent of λ DNA. We did not detect any increase in 3′3′-cGAMP production when λ DNA ligands were added in Mn^2+^-treated conditions (Fig. 2B). Similarly, under Mn^2+^-treated conditions, the addition of λ DNA also did not affect the catalytic activity of DncV, regardless of whether the protein concentration was altered or the reaction time was extended (Fig. 2C and D).

A recent study showed that bacteriophage RNA binds to the CdnE03 cyclase and promotes the synthesis of the CDN cGAMP to activate the CBASS immune response (34). To assess whether a similar mechanism exists for bacterial DncV, we supplemented reactions with various forms of ligand, including λ DNA, dsDNA, ssDNA, bacterial RNA, bacterial DNA, and T7 DNA, under both Mg^2+^- and Mn^2+^-treated conditions. The results indicated that under conditions treated with Mg^2+^, the addition of bacterial RNA and T7 DNA ligand inhibits the generation of products to varying degrees (Fig. 2E). Interestingly, in the Mn^2+^-mediated reaction system, the production of 3′3′-cGAMP is unaffected regardless of which ligand is added (Fig. 2F), indirectly suggesting that the activation of DncV by manganese ions does not require the participation of ligands.

### Mn^2+^ is preferentially used by DncV to produce cGAMP

**Fig 3:**
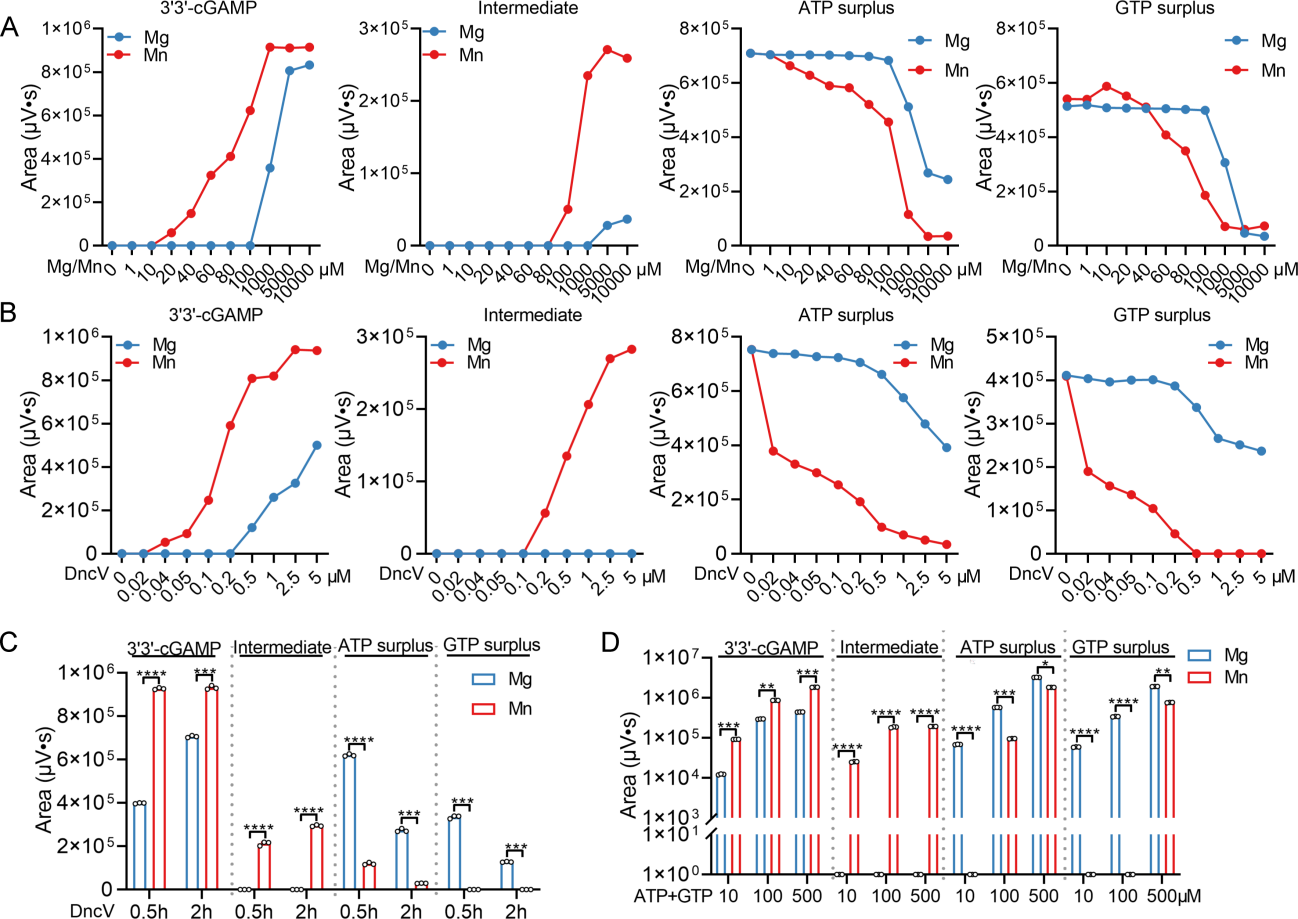
Mn^2+^ boosts DncV’s capacity to produce 3'3'-cGAMP. (**A**) Changing the Mg^2+^ or Mn^2+^ concentration (from 0 to 10,000 µM), 2 µM protein DncV was added into the reaction system (50 mM Tris-HCl, pH 7.5, MgCl_2_ or MnCl_2_, 100 µM ATP, and 100 µM GTP) for 30 min at 37°C. Next, all reaction mixtures were heated for 10 min at 98°C and centrifuged for 10 min at 12,000 rpm to obtain supernatant. Lastly, all samples were detected by HPLC. The production levels of 3'3'-cGAMP and intermediate products, as well as the remaining level of ATP and GTP, in the Mg^2+^ or Mn^2+^-mediated reaction system, were quantitatively analyzed using Empower software. (**B**) Changing the protein concentration (from 0 to 5 µM), different concentrations of the protein DncV were added into the reaction system for 30 min at 37°C. All other manipulations were as above. (**C**) 2 µM DncV was added into the reaction system for 0.5 h or 2 h at 37°C. All other manipulations were as above. (**D**) Changing the substrate concentration (from 10 to 500 µM), 2 µM protein DncV was added into the reaction system for 30 min at 37°C. All other manipulations were as above. Data represent the mean ± SEM of three biological replicates, each of which was performed with three technical replicates. *P*  values were calculated using one-way analysis of variance (ANOVA) for multiple comparisons. *P < 0.05; **P < 0.01; ***P < 0.001; ****P < 0.0001.

To dissect the influence of Mg^2+^ and Mn^2+^ concentrations on DncV activity, we first quantified 3′3′-cGAMP production and examined the consumption of ATP and GTP substrates. To examine how varying concentrations of Mn^2+^ or Mg^2+^ influence DncV-mediated 3′3′-cGAMP synthesis, we conducted enzymatic reactions using ion concentrations ranging from 0 to 10,000 µM. Across this range of ion concentrations, both Mg^2+^ and Mn^2+^ supported 3′3′-cGAMP synthesis, but Mn^2+^ consistently led to higher yields at lower concentrations. When Mn^2+^ served as the cofactor, maximal 3′3′-cGAMP production was achieved at 1,000 µM. By contrast, an approximately 10-fold higher concentration (10,000 µM) of Mg^2+^ was needed to reach similar 3′3′-cGAMP levels (Fig. 3A). This demonstrates that Mn^2+^ is substantially more efficient in facilitating DncV’s catalytic function. Moreover, the intermediate product in the cGAMP synthesis pathway appeared at much lower Mn^2+^ concentrations (100 µM) than were needed with Mg^2+^ (5,000 µM), suggesting that Mn^2+^ accelerates the reaction progression to the final product. Correspondingly, substrate consumption patterns reflected this enhanced efficiency. At equivalent or lower metal ion concentrations, Mn^2+^-mediated reactions depleted ATP and GTP more quickly, leaving less surplus of these nucleotides compared to Mg^2+^ conditions (Fig. 3A).

Next, we examined how varying DncV protein concentrations affected the reaction outcome under fixed metal ion conditions (Fig. 3B). With Mn^2+^ present, as little as 0.04 µM DncV was sufficient to detect 3′3′-cGAMP formation, and maximum production was achieved at 2.5 µM DncV. In contrast, at least 0.5 µM DncV was required to observe any cGAMP synthesis when Mg^2+^ was used, and even at 5 µM DncV, the reaction failed to reach saturation. Additionally, the intermediate product was readily detected at 2.5 µM DncV in the Mn^2+^-containing reaction, whereas no intermediate accumulation occurred under Mg^2+^ conditions. Analysis of ATP and GTP levels revealed that Mn^2+^-containing reactions consumed these substrates more thoroughly at lower DncV concentrations. In contrast, under Mg^2+^ conditions, substantial amounts of ATP and GTP remained, reflecting a less efficient catalytic turnover (Fig. 3B). Similar to DncV, DncE also exhibits higher product formation and substrate consumption in the Mn^2+^-mediated reaction system compared to the Mg^2+^-mediated system (Fig. S5).

To further elucidate the kinetic differences in DncV enzymatic activity when using Mg^2+^ or Mn^2+^ as cofactors, we monitored 3′3′-cGAMP production, intermediate formation, and substrate utilization over time (Fig. 3C). At 0.5 h, the Mn^2+^-containing reactions had already produced a substantial amount of 3′3′-cGAMP, reaching near-maximal levels. In contrast, the Mg^2+^-based reactions continued to increase in 3′3′-cGAMP yield and only achieved their peak production after 2 h. This rapid product formation under Mn^2+^ conditions indicates that Mn^2+^ accelerates the enzymatic turnover and enables DncV to reach maximum activity much sooner than with Mg^2+^. Notably, an intermediate product was observed exclusively in the Mn^2+^-mediated reaction and was detected at both 0.5 and 2 h, implying that Mn^2+^ not only speeds up the formation of 3′3′-cGAMP but also stabilizes or promotes the accumulation of transient intermediates along the reaction pathway. In contrast, no intermediate accumulated under Mg^2+^ conditions, suggesting a less efficient catalytic progression (Fig. 3C). The patterns of substrate consumption paralleled these observations. With Mn^2+^ present, ATP and GTP were rapidly depleted within 0.5 h, reflecting the enzyme’s enhanced catalytic efficiency and swift conversion of substrates into final products and intermediates. Under Mg^2+^ conditions, however, the substrate depletion occurred at a slower pace, indicating a lower overall reaction rate. Even after 2 h, the Mg^2+^-mediated reaction did not fully match the level of substrate consumption or product formation seen in the Mn^2+^ reaction after just 0.5 h (Fig. 3C). In summary, these time-course results demonstrate that Mn^2+^ significantly accelerates DncV’s kinetics, allowing for faster 3′3′-cGAMP synthesis, intermediate accumulation, and more rapid ATP/GTP utilization compared to the slower, less efficient kinetics observed under Mg^2+^ conditions.

To evaluate how different substrate concentrations affect DncV’s activity under Mn^2+^ or Mg^2+^ conditions, we measured 3′3′-cGAMP production and tracked the remaining levels of ATP and GTP at multiple input substrate concentrations (10, 100, and 500 µM) (Fig. 3D). As the initial substrate concentrations of ATP and GTP increased, both Mn^2+^- and Mg^2+^-based reactions produced more 3′3′-cGAMP, confirming that substrate availability positively influences reaction output. However, at all tested substrate concentrations, reactions containing Mn^2+^ generated significantly higher amounts of 3′3′-cGAMP compared to those with Mg^2+^. This indicates that Mn^2+^ not only enhances the catalytic turnover under standard conditions but continues to do so as substrate levels are raised. Moreover, the presence of Mn^2+^ facilitated the detection of intermediate products even at relatively modest substrate concentrations (100 µM), whereas such intermediates were barely detected or absent under Mg^2+^ conditions. This suggests that Mn^2+^-activated DncV can more efficiently convert starting materials through its reaction pathway, accumulating transient intermediates at lower thresholds of substrate input. Analysis of the remaining substrate levels further underscored the efficiency of Mn^2+^. At comparable ATP and GTP inputs, Mn^2+^ conditions led to markedly lower residual amounts of ATP and GTP than those observed in Mg^2+^ reactions. For instance, when starting with 500 µM ATP/GTP, the Mg^2+^-treated reaction left a substantial surplus of both substrates, whereas the Mn^2+^-treated reaction consumed a larger fraction of these nucleotides. Thus, Mn^2+^ conditions support a reaction environment in which the enzyme more rapidly and comprehensively utilizes available ATP and GTP to generate final and intermediate products. In summary, these results demonstrate that Mn^2+^ consistently improves DncV’s catalytic performance across a range of substrate concentrations (Fig. 3D).

### Mn^2+^ stimulates off-pathway product formation under ATP-rich conditions

Under physiological conditions, ATP concentrations often exceed GTP concentrations by approximately fivefold (35). To investigate how Mn^2+^ affects DncV-mediated nucleotide cyclization under physiologically relevant ATP: GTP ratios, we examined the enzyme’s activity with a 5:1 ATP-to-GTP substrate composition (Fig. S6). At 50 µM ATP and 10 µM GTP, adding Mn^2+^ not only increased the yield of 3′3′-cGAMP but also induced the formation of a second product, c-di-AMP, which was not observed in Mg^2+^-based reactions. This indicates that Mn^2+^ can drive off-pathway cyclization events, leading to the accumulation of c-di-AMP alongside the main product, 3′3′-cGAMP (Fig. S6A and B). To further dissect this phenomenon, we increased substrate concentrations to 500 µM ATP and 100 µM GTP (Fig. S6C). Under these conditions, Mn^2+^ treatment increased 3′3′-cGAMP production compared to Mg^2+^-treated reactions threefold. In addition, c-di-AMP levels rose by more than sevenfold in the presence of Mn^2+^. This disproportionate increase in c-di-AMP strongly supports the idea that Mn^2+^ promotes off-pathway cyclization, consuming additional substrates to generate c-di-AMP. Correspondingly, ATP and GTP were depleted more rapidly under Mn^2+^ conditions, reflecting increased overall enzymatic turnover and substrate utilization, including diversion into off-pathway products. Thus, while Mn^2+^ enhances the primary reaction, it also promotes an alternative cyclization pathway, generating off-pathway products like c-di-AMP.

**Fig 4:**
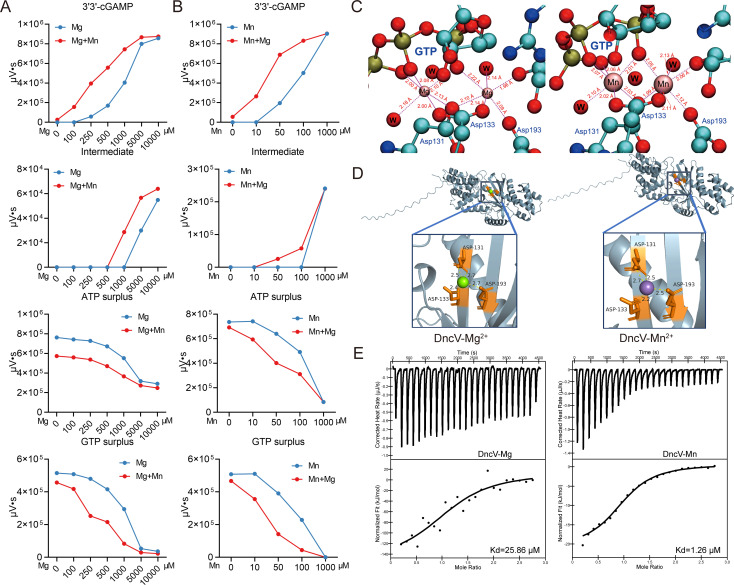
Mn^2+^ has a stronger binding affinity for DncV. (**A**) By adding the different concentrations of Mg^2+^ (from 0 to 10,000 µM), without or with 10 µM Mn^2+^, 2 µM protein DncV was added into the reaction system (50 mM Tris-HCl, pH 7.5, MgCl_2_, 100 µM ATP, and 100 µM GTP) for 30 min at 37°C. Next, all reaction mixtures were heated for 10 min at 98°C and centrifuged for 10 min at 12,000 rpm to obtain supernatant. Lastly, all samples were detected by HPLC. The production levels of 3'3'-cGAMP and intermediate products, as well as the remaining levels of ATP and GTP, were quantitatively analyzed using Empower software. (**B**) By adding the different concentrations of Mn^2+^ (from 0 to 1,000 µM), without or with 250 µM Mg^2+^, 2 µM protein DncV was added into the reaction system for 30 min at 37°C. All other manipulations were as above. The production levels of 3'3'-cGAMP and intermediate products, as well as the remaining levels of ATP and GTP, were quantitatively analyzed using Empower software. Data represent the mean ± SEM of three biological replicates, each of which was performed with three technical replicates. (**C**) A predictive model for the coordination relationship between Mn^2+^ and DncV. Left: schematic diagram of Mg^2+^ interaction with the amino acid residues of DncV. Right: schematic diagram of Mn^2+^ interaction with the amino acid residues of DncV. The purple dotted line indicates coordination, the red color indicates the ligand action distance, and the blue color indicates amino acid residues in the protein. (**D**) The molecular docking model of the DncV-Mg^2+^ or DncV-Mn^2+^ complex, prepared using PyMOL. Mg^2+^ is shown as a green sphere, and Mn^2+^ is shown as a purple sphere. Numbers accompanying dashed yellow lines represent the interaction distance. (**E**) The binding between Mg^2+^ or Mn^2+^ and DncV was examined with ITC. Data were analyzed using the Nano Analyze software.

Given that both Mg^2+^ and Mn^2+^ coexist within cells, we then explored their combined effects. Adding Mn^2+^ to a Mg^2+^-activated DncV reaction at the same Mg^2+^ concentration led to higher product yields and increased substrate consumption (Fig. 4A). Thus, Mn^2+^ can further enhance DncV activity already supported by Mg^2+^, accelerating the reaction and reducing substrate surplus. In contrast, when Mg^2+^ was added to an Mn^2+^-activated system, the resulting product yield plateaued at about 1 mM Mn^2+^, even as Mg^2+^ was increased (Fig. 4B). This suggests that once Mn^2+^ has fully activated DncV, adding Mg^2+^ cannot further improve enzyme efficiency or increase substrate consumption (Fig. 4B). At saturating Mn^2+^ concentrations, the reaction has effectively reached its maximum capacity for product formation. Consistent with these observations, similar trends were observed when examining the *E. coli* homolog CdnE, reinforcing that the synergistic yet ultimately Mn^2+^-dominated activation dynamics might be a general feature of bacterial dinucleotide cyclases (Fig. S7A and B).

In summary, these results demonstrate that Mn^2+^ not only enhances the primary cGAMP-producing activity of DncV but also drives off-pathway product formation (c-di-AMP) under physiologically relevant ATP:GTP ratios. While Mn^2+^ can amplify the effect of Mg^2+^ to increase both product formation and substrate consumption, the Mg^2+^ addition could not improve a Mn^2+^-saturated reaction.

### Mn^2+^ has a higher affinity with DncV compared with Mg^2+^

To elucidate why Mn^2+^ enhances DncV activity more effectively than Mg^2+^, we carried out detailed computational analyses and performed high-level quantum chemical interaction energy calculations using the crystal structure of DncV (PDB ID: 4U03). After optimizing the structure at the PBE0-D3/def2-TZVP level, we computed the interaction energies between each metal ion (Mg^2+^ or Mn^2+^) and the protein’s active site, which includes GTP and key coordinating amino acid residues. The calculations revealed that substituting Mg^2+^ with Mn^2+^ significantly improved the metal ion’s binding affinity within the active center (Fig. 4C). Specifically, the binding energy for Mg^2+^ was determined to be −3120.2330 kcal/mol. Upon replacing Mg^2+^ with Mn^2+^, the binding energy became even more favorable at −3352.2650 kcal/mol (Table 1). This enhancement in binding strength strongly indicates that Mn^2+^ forms a more stable and tightly bound complex with DncV than Mg^2+^.

**TABLE 1:** Interaction energies between metal ions (Mg^2+^ and Mn^2+^) and protein amino acids

System	*E*_complex_ (×10^3^ kcal/mol)	*E*_protein_ (×10^3^ kcal/mol)	*E*_Mg/Mn_ (×10^3^ kcal/mol)	*E*_BSSE_ (kcal/mol)	Δ*E* (kcal/mol)
Mg^2+^	−5662.6497	−5409.9664	−249.5361	27.0701	−3120.2330
Mn^2+^	−6855.6819	−5409.9507	−1442.3557	23.1839	−3352.2650

To probe the structural origins of this difference, we examined the optimized coordination environments of Mg^2+^ or Mn^2+^ (Fig. 4D). In both cases, the metal ion (Mg^2+^ or Mn^2+^) coordinates to the phosphate oxygens of GTP, surrounding water molecules, and the carboxyl groups of conserved aspartate residues (Asp131, Asp133, and Asp193), establishing a stable configuration crucial for catalysis. While the types of ligands remain the same, the Mn^2+^ complex exhibits generally shorter metal-ligand distances compared to the Mg^2+^ complex. These more compact interactions strengthen the overall binding, thereby increasing the stability of the Mn^2+^-protein complex. The isothermal titration calorimetry (ITC) experimental results further confirm this conclusion: compared with Mg^2+^ (Kd = 25.86 µM), Mn^2+^ has a stronger binding affinity to DncV (Kd = 1.26 µM) (Fig. 4E). Consistently, the ITC assay with DncE produced essentially identical results (Fig. S7C). Moreover, the DncV-ATP-GTP-Mn^2+^ complex exhibits a shorter metal-ligand distance as compared to the Mg^2+^ bound complex (Fig. S8A). In addition, pre-incubation of the DncV with Mn^2+^ can even further enhance the DncV’s affinity for ATP or GTP (Fig. S8B and C). In contrast, Mg^2+^ does not alter the DncV’s binding capacity with the substrate, whether ATP or GTP (Fig. S8B and C). In summary, the computational results, structural analysis, and ITC results show that Mn^2+^ achieves a higher binding affinity in DncV’s active site due to its ability to form tighter interactions with substrate (ATP and GTP) and the coordinating amino acids. This enhanced affinity at the atomic level provides a mechanistic basis for why Mn^2+^ more effectively boosts the catalytic activity of DncV than Mg^2+^.

### Mn^2+^ alleviates the inhibition of DncV activity by folate

**Fig 5:**
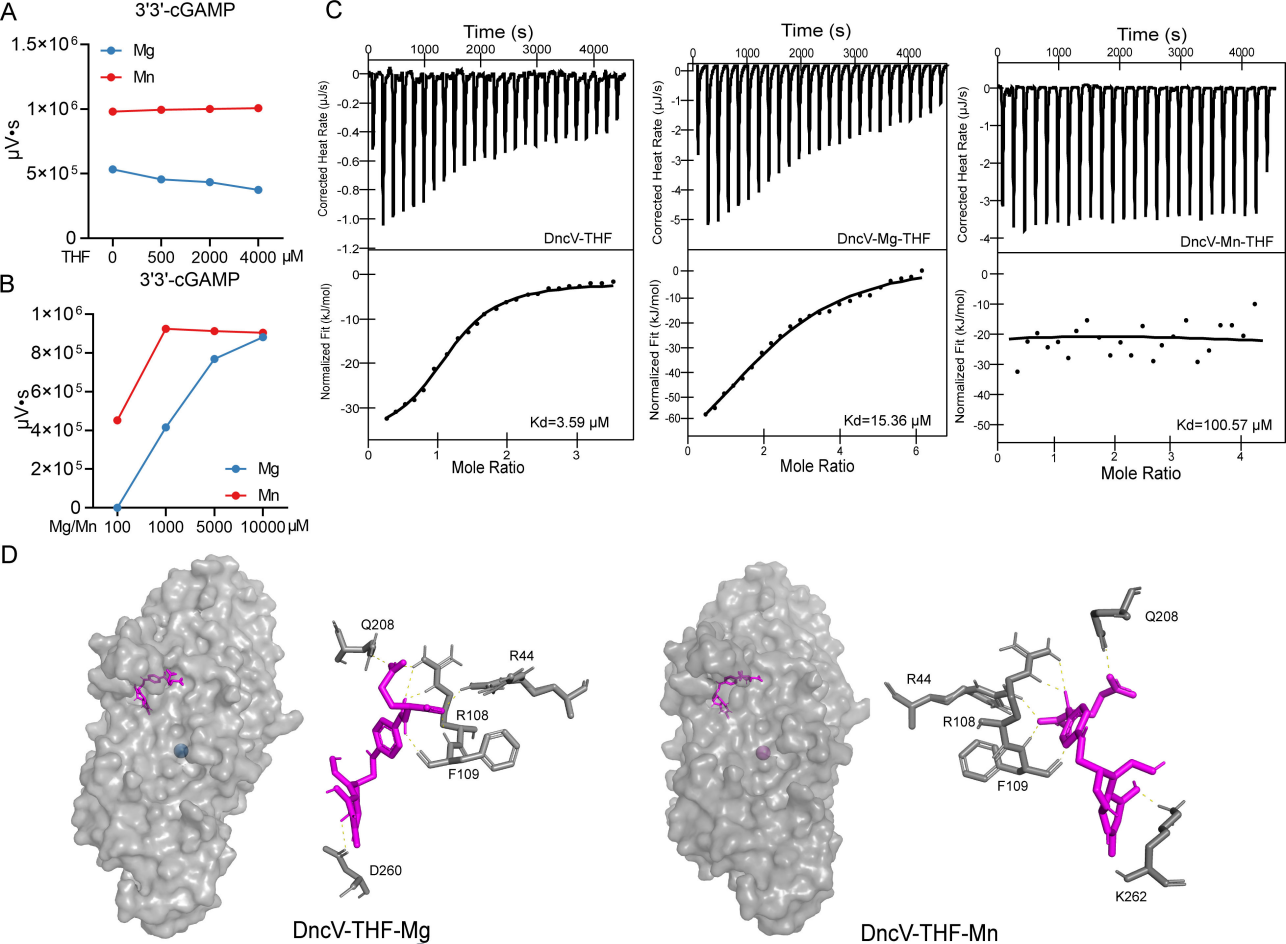
Mn^2+^ can alleviate folate-mediated inhibition of DncV. (**A**) Adding different concentrations of THF (from 0 to 4,000µM), the amount of 3'3'-cGAMP production under Mg^2+^- or Mn^2+^-treated reaction system. DncV was added into the reaction system (50mM Tris-HCl, pH 7.5, 1mM Mn^2+^ or Mg^2+^, different concentrations of THF, 100µM ATP, and 100µM GTP) for 30min at 37°C. The mixtures were heated and centrifuged to obtain supernatants. All samples were detected by HPLC. The analysis and summary were conducted using the software integrated with the HPLC system. (**B**) Adding 500µM THF, added different concentrations of Mn^2+^ or Mg^2+^, the amount of 3'3'-cGAMP production under Mg^2+^- or Mn^2+^-treated reaction system. Data represent the mean ± SEMof three biological replicates, each of which was performed with three technical replicates. (**C**) The binding of THF to DncV, DncV-Mg^2+^, and DncV-Mn^2+^ was examined by ITC. Data were analyzed using the Nano Analyze software. (**D**) Molecular docking model of the DncV-THF complex with Mg^2+^ or Mn^2+^. The three-dimensional structures of the protein DncV are shown in gray. DncV was displayed in a similar orientation in DncV-THF-Mg^2+^ or Mn^2+^ models. THF was shown as purple sticks. The pyMOL software was used to visualize 3D structures of ligand-amino acid residue interactions, with potential hydrogen bonds indicated by yellow dashed lines.

It has been reported that folate-like molecules like 5-methyltetrahydro folic acid (5MTHF), 5-methyltetrahydrofolate diglutamate (5MTHFGLU2), tetrahydrofolate (THF), or folic acid (FA) can reduce DncV-mediated cGAMP synthesis (36). To investigate whether Mn^2+^ can mitigate the known inhibitory effect of folate-like molecules on DncV activity, we measured 3′3′-cGAMP production under varying conditions of tetrahydrofolate (THF) and divalent ions (Mg^2+^ or Mn^2+^). We first confirmed that the purified apo DncV enzyme, which does not bind 5MTHF/5MTHFGLU2, was nonetheless sensitive to inhibition by THF in a Mg^2+^-based reaction system (Fig. 5A). As the THF concentration increased, 3′3′-cGAMP production steadily declined, demonstrating that in a Mg^2+^ environment, folate-like molecules effectively impair DncV’s catalytic function. In striking contrast, when Mn^2+^ replaced Mg^2+^ as the cofactor, the inhibitory effect of THF on DncV activity was largely abolished (Fig. 5A). Even at higher THF concentrations, DncV maintained robust 3′3′-cGAMP production under Mn^2+^ conditions, indicating that Mn^2+^ confers a protective effect against folate-mediated inhibition.

To further delineate the metal ion’s capacity to overcome folate inhibition, we varied both the metal ion and THF concentrations. When the Mg^2+^ concentration was kept at a low level (100 µM), DncV activity was completely suppressed by 500 µM THF. However, substituting Mn^2+^ for Mg^2+^ restored a substantial level of 3′3′-cGAMP synthesis under these same conditions (Fig. 5B). Additionally, achieving maximal cGAMP production in the presence of THF required a very high concentration (10,000 µM) of Mg^2+^, while only 1,000 µM of Mn^2+^ was needed to reach a comparable level of enzyme activity (Fig. 5B). This finding underscores the superior ability of Mn^2+^ to outcompete the inhibitory influence of THF relative to Mg^2+^. In summary, these results demonstrate that Mn^2+^ effectively alleviates the inhibitory effect of folate-like molecules on DncV activity. Compared to Mg^2+^, Mn^2+^ allows DncV to maintain a high level of 3′3′-cGAMP production even under the conditions of folate-mediated inhibition, suggesting that Mn^2+^ uniquely stabilizes or enhances DncV’s active state in the presence of inhibitory ligands.

To further elucidate why Mn^2+^ can relieve the inhibitory effects of folate-like molecules, we measured the binding affinity between the DncV without or with metal ions and THF by using ITC. The results show that DncV exhibits significant binding affinity toward THF, with an affinity constant of 3.59 µM (Fig. 5C, left). Upon further investigation, when DncV was pre-incubated with metal ions (Mg^2+^ or Mn^2+^) before assessing the binding affinity of the DncV-metal ion complexes with THF, we observed that the presence of Mn^2+^ significantly reduced the affinity between the DncV and THF (Fig. 5C, middle). In contrast, the affinity remained virtually unchanged in the presence of Mg^2+^ (Fig. 5C, right). Further structural analysis revealed an intriguing phenomenon: regardless of which metal ion is present (Mg^2+^ or Mn^2+^), the binding site of the DncV with THF remained virtually identical (Fig. 5D). However, the number of hydrogen bonds formed between the DncV and THF varied significantly. Specifically, in the DncV-THF-Mg^2+^ complex, eight hydrogen bonds were formed between the protein and THF (Fig. 5D, left), whereas in the DncV-THF-Mn^2+^ complex, only seven hydrogen bonds were observed (Fig. 5D, right). This finding further elucidates why the inhibitory effect of THF on DncV is reduced in the presence of manganese ions. These findings suggest that Mn^2+^ can partially mitigate the inhibitory effects of folate on the protein.

### Mn^2+^ activates the DncV-CapV cascade without affecting CapV activity

**Fig 6:**
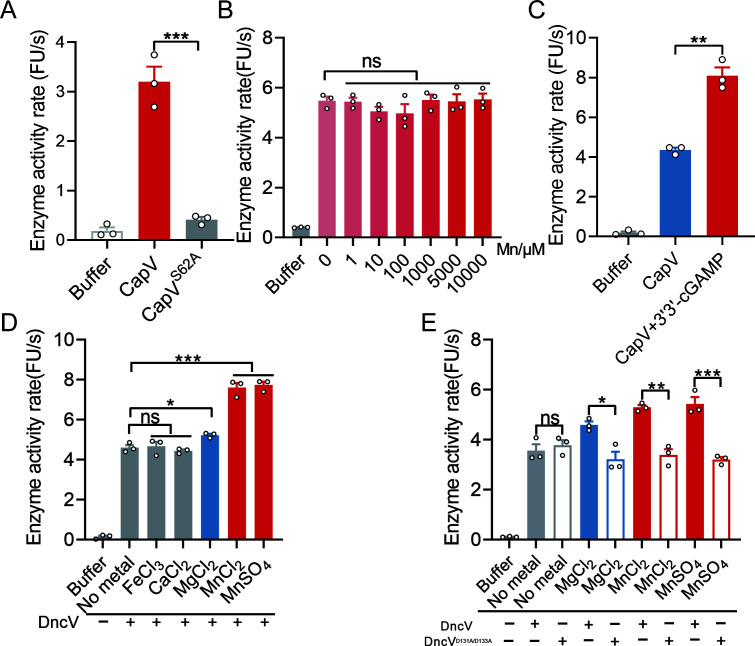
Mn^2+^ can indirectly enhance the enzymatic activity of phospholipase CapV. (**A**) The phospholipase activity of the proteins CapV and CapV^S62A^ was detected using the fluorescent substrate Resorufin butyrate. (**B**) Mn^2+^ cannot directly enhance the enzymatic activity of the CapV. Different concentrations of Mn^2+^ (from 0 to 10,000 µM) were added to the CapV reaction system and incubated, followed by the detection of its enzyme activity. The enzymatic activity of the CapV was assayed at a constant temperature of 37°C with an excitation wavelength of 550 nm and an emission wavelength of 591 nm. (**C**) 3'3'-cGAMP can enhance the enzymatic activity of the phospholipase CapV. 3'3'-cGAMP was added to the CapV reaction system and incubated, followed by the detection of its enzyme activity. (**D**) Mn^2+^ indirectly enhances the enzymatic activity of the CapV through DncV. DncV was added into the reaction system (50 mM Tris-HCl, pH 7.5, different metal ions, 100 µM ATP, and 100 µM GTP) for 30 min at 37°C. The mixtures were heated and centrifuged to obtain supernatants. All supernatants were added in appropriate amounts to the CapV reaction system and incubated, followed by the detection of CapV enzyme activity. (**E**) DncV^D131A/D133A^ cannot enhance the enzymatic activity of the CapV. DncV or DncV^D131A/D133A^ was added into the reaction system (50 mM Tris-HCl, pH 7.5, 1 mM Mn^2+^ or Mg^2+^, 100 µM ATP, and 100 µM GTP) for 30 min at 37°C. The mixtures were heated and centrifuged to obtain supernatants. All supernatants were added in appropriate amounts to the CapV reaction system and incubated, followed by the detection of CapV enzyme activity. Data represent the mean ± SEM of three biological replicates, each of which was performed with three technical replicates. *P*  values were calculated using a two-tailed Student’s *t*-test for paired comparisons or a one-way analysis of variance (ANOVA) for multiple comparisons. *P < 0.05; **, *P* < 0.01; ***P < 0.001; ns, not significant.

Phospholipase CapV has been reported to be essential for anti-phage (37), and this was further verified by our experiments (Fig. 6; Fig. S9). We confirmed that CapV has phospholipase activity (Fig. 6A). Thus, it is reasonable to speculate that Mn^2+^ also possesses the ability to enhance the phospholipase activity of CapV. To determine whether Mn^2+^ directly influences the phospholipase activity of CapV, we first tested purified CapV in the presence of increasing Mn^2+^ concentrations. As shown in Fig. 6B, even at higher Mn^2+^ levels, there was no significant change in CapV’s enzymatic activity rate. This indicates that Mn^2+^ does not directly activate CapV. Because prior studies have indicated that 3′3′-cGAMP, synthesized by DncV, can stimulate CapV phospholipase activity (37), our experimental results also confirm this conclusion (Fig. 6C), we hypothesized that Mn^2+^ might indirectly enhance CapV function by boosting DncV-mediated 3′3′-cGAMP production. To test this, we performed DncV reactions under different metal ion conditions and then incubated the resulting supernatants with CapV. The results revealed that while supernatants from Fe³^+^ and Ca²^+^ reactions failed to enhance CapV activity, both Mg^2+^- and Mn^2+^-treated DncV supernatants did so. Notably, the Mn^2+^-treated DncV reaction mixture elicited a stronger enhancement of CapV activity than the Mg^2+^-treated mixture (Fig. 6D). This finding strongly supports the notion that Mn^2+^ indirectly augments CapV’s phospholipase activity by increasing the level of 3′3′-cGAMP produced by DncV. Further genetic evidence confirms that this effect is dependent on functional DncV and CapV. Mutating the catalytic residues in DncV (D131A/D133A), which abolishes its ability to generate 3′3′-cGAMP, resulted in no increase in CapV activity (Fig. 6E). Similarly, the S62A mutation in CapV (CapV^S62A^) abolished its phospholipase activity (Fig. 6A), demonstrating that both an active DncV and an intact CapV catalytic center are required for the observed activation. In summary, these results demonstrate that Mn^2+^ enhances the activity of phospholipase CapV not directly by affecting the enzyme itself, but rather indirectly by increasing the production of 3'3'-cGAMP through DncV activation. This enhancement occurs through Mn^2+^-mediated activation of DncV, leading to increased levels of 3'3'-cGAMP, which subsequently stimulates CapV activity.

### Mn^2+^ is required for the antiviral potential of the CBASS system

The CBASS system is essential for the anti-phage potential of bacteria (8, 38). Mn^2+^ plays an important role in innate immunity and enhances the host antiviral function (16, 19, 39). We also noticed the intracellular Mn^2+^ and Mg^2+^ concentrations during T4 and P1 infection, and the results showed that both metal ions were accumulated during phage infection in bacterial cells (Fig. S10).

**Fig 7:**
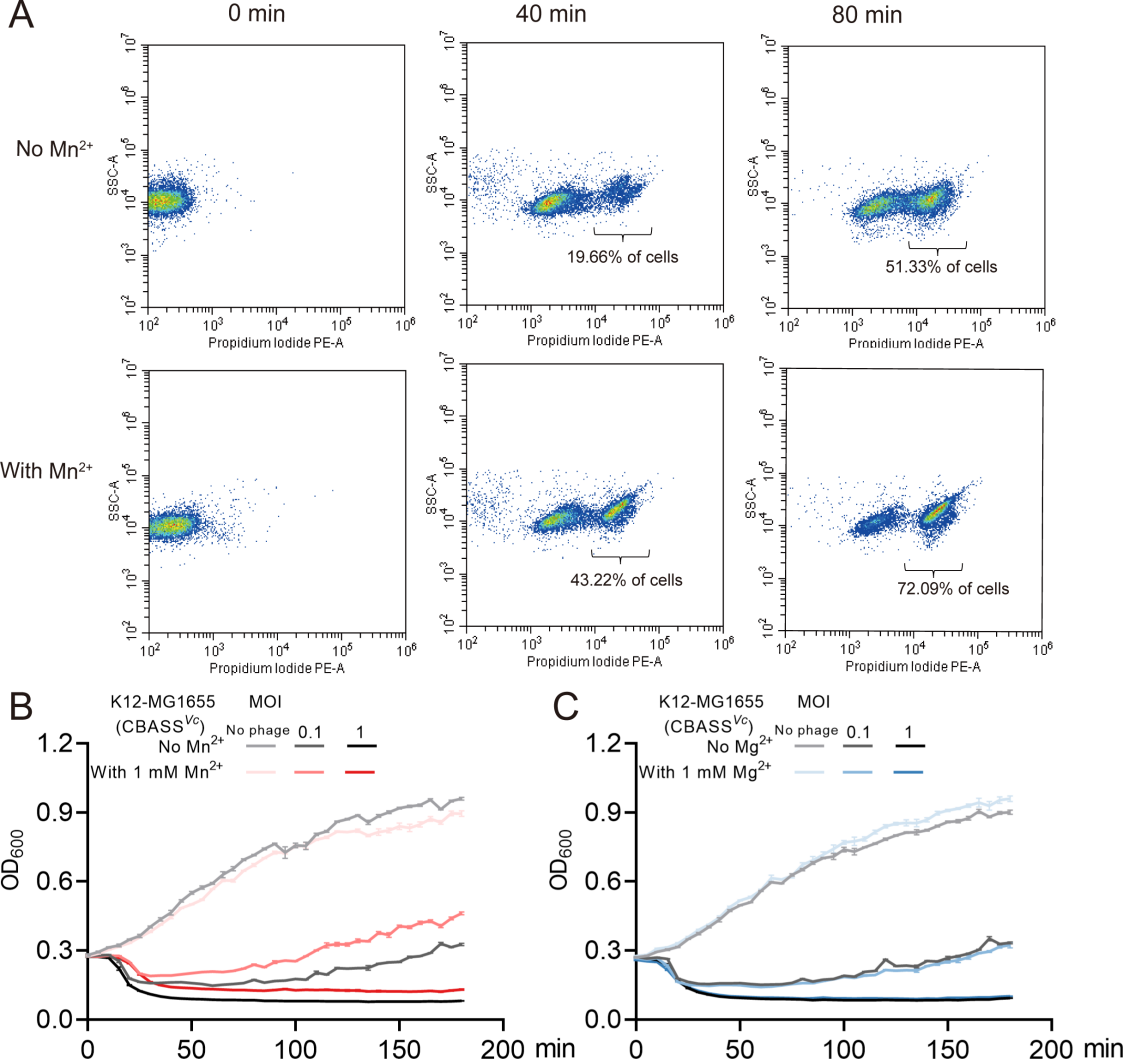
Elevating intracellular levels of Mn^2+^ bolsters the CBASS system’s capability to fend off phage infections. (**A**) In the presence of Mn^2+^, strains containing the CBASS system undergo premature "suicide" upon phage infection. K12-MG1655 containing CBASS system (no Mn^2+^/with 1 mM Mn^2+^) was stained with propidium iodide, a fluorescent DNA-binding agent that penetrates cells that have impaired membrane integrity. Cells were infected by phage P1 (MOI = 2), and fluorescence intensity was measured at 0 min, 40 min, and 80 min. This experiment was performed with three biological replicates with triplicate technical replicates. (**B**) Growth curves in liquid culture for K12-MG1655 (CBASS*^Vc^*) (no Mn^2+^/1 mM Mn^2+^) infected by phage P1 at 37°C. Bacteria were infected at time = 0 at an MOI of 0, 0.1, or 1. (**C**) Growth curves in liquid culture for K12-MG1655 (CBASS*^Vc^*) (no Mg^2+^/1 mM Mg^2+^) infected by phage P1 at 37°C. Bacteria were infected at time = 0 min at an MOI of 0, 0.1, or 1. OD_600_, optical density at a wavelength of 600 nm. Data represent the mean ± SEM of three biological replicates, each of which was performed with three technical replicates.

To assess the role of Mn^2+^ in boosting the antiviral activity of the CBASS, we first monitored bacterial cell death during phage infection in the presence and absence of Mn^2+^. Utilizing a *V. cholerae*-derived CBASS system in an *E. coli* K12-MG1655 (CBASS*^Vc^*) strain infected with phage P1, we observed that the addition of Mn^2+^ significantly accelerated the onset of bacterial cell death (Fig. 7A). At 40 min post-infection, approximately 43.22% of cells were dead in the Mn^2+^-treated sample, compared to 19.66% in the untreated control. By 80 min, the mortality rate increased to 72.09% with Mn^2+^, versus 51.33% without Mn^2+^. These findings suggest that Mn^2+^ enhances CBASS-mediated cell killing, thereby triggering an earlier and more robust self-destruct response in infected bacteria (Fig. 7A).

Next, we compared bacterial growth curves under phage challenge with and without Mn^2+^ supplementation. In the presence of Mn^2+^, the K12-MG1655 (CBASS*^Vc^*) strain showed improved growth and greater resistance to P1 infection, indicating that Mn^2+^ not only promotes faster CBASS activation but also contributes to overall enhanced survival and growth in the face of phage attack (Fig. 7B). In contrast, the addition of Mg^2+^ did not produce a similar protective effect, underscoring the specificity of Mn^2+^ in strengthening CBASS-mediated defense (Fig. 7C).

**Fig 8:**
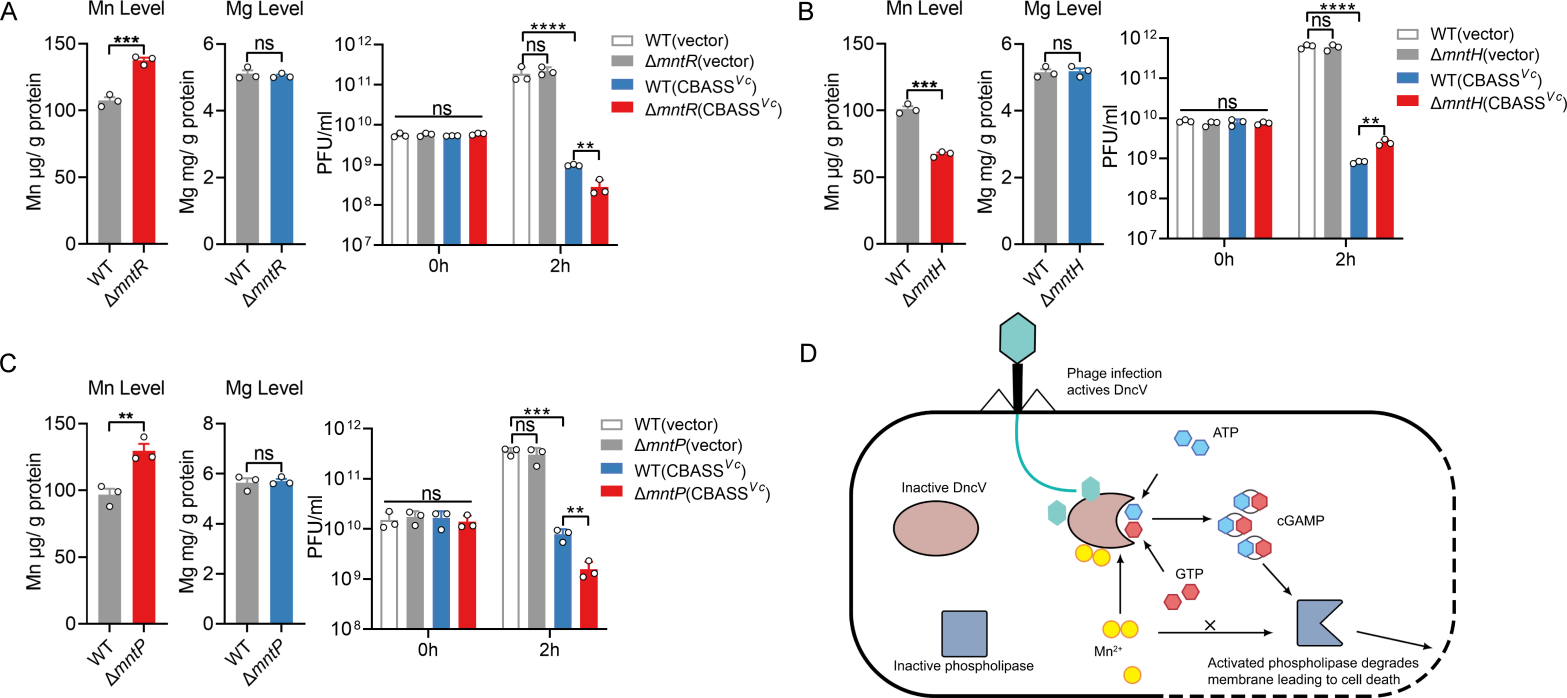
Intracellular Mn^2+^ homeostasis in bacteria mediates the host’s antiphage immune response efficacy through CBASS activity. (**A**) The intracellular concentration of Mn^2+^ or Mg^2+^ associated with WT or Δ*mntR* was measured by ICP-MS. K12-MG1655 (vector), K12-MG1655 (CBASS*^Vc^*), Δ*mntR* (vector), and Δ*mntR* (CBASS*^Vc^*) were infected by P1 (MOI = 0.1), and phage titer was measured at 0 h and 2 h. (**B**) The intracellular concentration of Mn^2+^ or Mg^2+^ associated with WT or Δ*mntH* was measured by ICP-MS. K12-MG1655 (vector), K12-MG1655 (CBASS*^Vc^*), Δ*mntH* (vector), and Δ*mntH* (CBASS*^Vc^*) were infected by P1 (MOI = 0.1), and phage titer was measured at 0 h and 2 h. (**C**) The intracellular concentration of Mn^2+^ or Mg^2+^ associated with WT or Δ*mntP* was measured by ICP-MS. K12-MG1655 (vector), K12-MG1655 (CBASS*^Vc^*), Δ*mntP* (vector), and Δ*mntP* (CBASS*^Vc^*) were infected by P1 (MOI = 0.1), phage titer was measured at 0 h and 2 h. Data represent the mean ± SEM of three biological replicates, each of which was performed with three technical replicates. *P*  values were calculated using a two-tailed Student’s *t*-test for paired comparisons or a one-way analysis of variance (ANOVA) for multiple comparisons. **P < 0.01; ***P < 0.001; ****P < 0.0001; ns, not significant. (**D**) A model for Mn^2+^ enhances the anti-phage function of the CBASS system. Following phage infection, the intracellular concentration of Mn^2+^ increases significantly, and the expression of Mn^2+^ transport systems is upregulated. Mn^2+^ directly enhances the enzymatic activity of DncV, leading to increased production of the secondary messenger 3'3'-cGAMP. This, in turn, indirectly enhances the activity of the phospholipase CapV, further promoting bacterial membrane lysis and curtailing phage replication.

To further link increased intracellular Mn^2+^ levels with heightened antiviral defense, we employed the CRISPR-Cas9 system to generate three mutant strains in K12-MG1655 associated with the Mn^2+^ transport system, namely Δ*mntR*, Δ*mntH*, and Δ*mntP*. These three proteins are highly conserved among bacterial species (Fig. S11 through S13). The *mntR* is a vital gene encoding a Mn^2+^-responsive transcriptional regulator, the results show that deletion of mntR leads to a significant increase in intracellular manganese ion concentration (Fig. 8A). When tested against phage, the Δ*mntR* mutant harboring the CBASS system demonstrated stronger phage resistance, correlating with its elevated Mn^2+^ content (Fig. 8A). Moreover, in *E. coli* K12-MG1655, Mn^2+^ homeostasis is regulated by uptake transporters like MntH and export systems such as MntP, a dedicated manganese efflux pump that prevents intracellular toxicity under excess conditions (27, 29). Quantitative analysis using ICP-MS demonstrated that the intracellular Mn^2+^ concentration was significantly reduced in the Δ*mntH* mutant compared to the wild-type strain (Fig. 8B), whereas the Δ*mntP* mutant exhibited a marked increase in Mn²^+^ accumulation (Fig. 8C). To further investigate the molecular mechanism underlying the synergistic antiphage defense between Mn^2+^ homeostasis and the CBASS system, we electroporated Δ*mntH* and Δ*mntP* mutants with plasmids encoding the CBASS system and assessed their antiphage capacity. The results demonstrated that: Δ*mntH* (CBASS*^Vc^*) exhibited a significantly reduced survival rate against phage infection compared to the wild type (CBASS*^Vc^*) (Fig. 8B), suggesting that decreased intracellular Mn^2+^ levels may compromise the defensive efficacy of the CBASS system; in contrast, Δ*mntP* (CBASS*^Vc^*) displayed markedly enhanced antiphage activity (Fig. 8C), implying that Mn^2+^ overload might potentiate immune responses by activating the CBASS system. These results further support the idea that an increased intracellular Mn^2+^ concentration is associated with a stronger anti-phage function. Collectively, these results establish that Mn^2+^ enhances the antiviral function of the CBASS system. By promoting earlier cell death of infected bacteria, Mn^2+^ emerges as a crucial cofactor for optimizing bacterial innate immune defenses against phage infection (Fig. 8D).

## DISCUSSION

The ability of cells, both prokaryotic and eukaryotic, to sense infection and mount an appropriate immune-like response is of central importance for survival. The discovery of cyclic GMP-AMP synthase (cGAS) in mammalian cells highlighted the power of a nucleotidyltransferase that recognizes cytosolic DNA and synthesizes the second messenger 2'3'-cGAMP, ultimately activating STING to trigger antiviral and inflammatory pathways. More recently, it has become clear that analogous or homologous systems exist in other eukaryotes, like insects and in bacteria, typically referred to as cGAS-like or CD-NTase enzymes (1, 40). These bacterial systems, known as CBASS, respond to phage infection by destroying the infected cell before viral replication completes. Despite these parallels, the role of metal ions in these cGAS-like pathways has often been overshadowed. This and previous studies underscore that Mn^2+^ emerges as a key cofactor driving cGAMP synthesis across prokaryotic and eukaryotic systems (Fig. 1E and F) (19), suggesting a deep evolutionary thread that ties together innate immunity from bacteria to humans. In this study, we showed that during phage infection, intracellular Mn^2+^ levels increase and, in turn, promote the production of cyclic GMP-AMP (cGAMP) by activating bacterial cGAS (DncV, DncE). This elevated cGAMP subsequently activates the phospholipase CapV, driving an abortive infection phenotype to block phage replication. Importantly, we show that Mn^2+^ can override the inhibitory effects of folate-like molecules on DncV while expanding the possible substrate scope of this enzyme. Together, these results underscore a previously unappreciated role for Mn^2+^ in modulating bacterial innate immunity and provide new insights into the mechanistic parallels between bacterial and eukaryotic cGAS-STING pathways. Here, we highlight and discuss the major innovations arising from this work.

A key observation is that upon infection by both phage P1 and T4, *E. coli* upregulates Mn^2+^ transporter genes *mntH* and accumulates Mn^2+^ (Fig. 1A through 1D). This phenomenon suggests that bacterial cells have evolved a rapid metal-ion mobilization strategy in response to viral intrusion. Analogous “ion flux” events have been noted in eukaryotic systems, where viruses or bacteria can induce changes in Mn^2+^ or Zn^2+^ to modulate host defense pathways (19, 41), but the report of Mn^2+^ surging in phage-infected bacteria is new and striking. Our enzymatic assays clearly show that DncV is more than an order of magnitude more efficient with Mn^2+^ than with Mg^2+^ (Fig. 3). This allows DncV to generate high levels of 3'3'-cGAMP in a short time frame, triggering CapV and leading to an abortive infection phenotype that halts phage replication. Crucially, when we genetically or biochemically manipulated Mn^2+^ levels, the timing and strength of cell suicide changed correspondingly, confirming that Mn^2+^ is a master regulator of CBASS kinetics (Fig. 7A). We generated single-gene deletion mutants of *mntR*, *mntH,* and *mntP* in *E. coli* K12-MG1655 using the CRISPR-Cas9 system. ICP-MS analysis revealed that, compared to the wild-type strain, the Δ*mntH* mutant exhibited significantly reduced intracellular Mn^2+^ levels (Fig. 8B), while the Δ*mntR* and Δ*mntP* mutants showed marked Mn^2+^ accumulation exceeding wild-type levels (Fig. 8A and C). Intracellular Mg^2+^ concentrations remained largely unchanged across strains (Fig. 8A through 8C), indicating specificity for Mn^2+^ homeostasis. To link this to phage resistance, we electroporated the CBASS*^Vc^* plasmid (or empty vector control) into these mutants and performed phage titration assays. The results demonstrated that Δ*mntH* (CBASS*^Vc^*) had weaker anti-phage activity, whereas Δ*mntR* (CBASS*^Vc^*) and Δ*mntP* (CBASS*^Vc^*) displayed enhanced resistance compared to wild-type (CBASS*^Vc^*) (Fig. 8A through 8C). These findings directly establish that *mntR*-, *mntH*-, and *mntP*-dependent Mn^2+^ accumulation is causally required for robust CBASS-mediated defense during phage infection. Moreover, by artificially adding Mn^2+^ to cultures, we found it possible to fine-tune the antibacterial potency of the CBASS system (Fig. 7A and B), offering the potential for industrial or clinical applications wherein accelerated phage clearance is desirable. Evidently, the high conservation of MntH, MntR, MntP, and DncV across bacterial species (Fig. S4C, S11 to S13) suggests an evolutionarily conserved mechanism linking Mn^2+^ homeostasis to cyclic nucleotide immunity, extending our findings beyond *E. coli* and *V. cholerae*. We believe these additions greatly enhance the study’s value and provide a complete evolutionary context.

It has been recognized that mammalian cGAS is significantly potentiated by Mn^2+^ (19), with several studies showing that Mn^2+^ can lower the dsDNA threshold required to activate cGAS or even bypass dsDNA altogether under some conditions (20, 33). In these contexts, Mn^2+^-induced cGAS undergoes globally similar conformational changes that favor the production of the 2'3'-cGAMP (20). Similarly, we show that Mn^2+^ exerts a comparable “enhancer” effect on bacterial cGAS-like enzymes, accelerating the formation of 3′3′-cGAMP. Thus, Mn^2+^ stands out as a conserved cofactor across evolutionary space, capable of boosting cGAS or cGAS-like cyclases irrespective of whether the output is 2′3′-cGAMP or 3′3′-cGAMP.

Recent reviews emphasize that cGAS-STING-like pathways are pervasive across the tree of life, from vertebrates to invertebrates and even many bacteria (40). In vertebrates (including mammals), classical cGAS synthesizes 2′3′-cGAMP, which binds STING to trigger interferon, NF-κB, and autophagy responses. In Drosophila melanogaster, cGLR1 and cGLR2 produce 3′2′-cGAMP or a mixture of 2′3′- and 3′2′-cGAMP, activating STING- and NF-κB–based antiviral defenses (42). In bacteria, cGAS-like enzymes (CD-NTases) generate a diversity of cyclic di- and trinucleotides, from 3′3′-cGAMP to more exotic forms, which activate cell-killing effectors. In all these scenarios, the unifying theme is that a cyclic nucleotide is synthesized upon viral infection, leading to a protective outcome. A second strong parallel is emerging around Mn^2+^ as a potent cofactor that can amplify or refine cGAS-like enzymatic activity, although no experimental evidence has been reported in *Drosophila melanogaster*.

From an evolutionary vantage, these parallels point to a scenario in which the core architecture of cGAS-like nucleotidyltransferases coevolved with divalent cation usage, particularly favoring Mn^2+^ under certain stress or infection contexts. Because phages and viruses exert intense selective pressure, any mutation or strategy that allows more rapid or sensitive detection of invaders is advantageous. Mn^2+^’s ability to form shorter, stronger bonds with catalytic aspartates or glutamates in the enzyme’s active site likely contributed to the repeated evolution of Mn^2+^-enhanced cGAS-like pathways. The difference in linkage isomers (2′3′-, 3′3′-, 3′2′-cGAMP) could reflect the distinct structural features of each cGAS-like protein lineage while still retaining the unifying requirement for a potent metal cofactor.

In many prokaryotic contexts, Mg^2+^ is traditionally seen as the essential cation for polymerases, nucleases, and other phosphate-handling enzymes. Our findings, however, show that Mn^2+^ can transform the dynamics of cGAMP synthesis in CBASS. The upregulation of Mn^2+^ transporters (Fig. 1B; Fig. S2C) indicates that bacteria orchestrate the Mn^2+^ homeostasis under infection stress. This shift parallels certain eukaryotic responses, where Mn^2+^ is mobilized from organelles (mitochondria, Golgi, or specialized vesicles) to potentiate cGAS or STING (19).

In eukaryotic systems, the concept of “nutritional immunity” describes how hosts withhold essential metal ions, such as Mn^2+^, to weaken pathogenic bacteria. By reducing intracellular Mn^2+^ availability, host cells inhibit critical bacterial enzymes and defenses, thereby slowing or thwarting infection. Recent studies showed that phage anti-CBASS proteins can sequester cyclic trinucleotides and dinucleotides that counteract CBASS antiviral signaling (43, 44). Future studies might reveal whether certain phages have evolved to sabotage Mn^2+^ import or sequester metals, akin to “nutritional immunity” strategies found in eukaryotes or the Viral sponges to sequester Mn^2+^.

One of the most tangible outcomes of heightened Mn^2+^ levels is the early onset of cell death in infected bacteria (Fig. 7A). By driving DncV to flood the cell with cGAMP and rapidly activate CapV phospholipase, the bacterium effectively chooses to save the population by sacrificing itself. This abortive infection phenomenon is not new; it underpins many toxins or small-molecule-based defenses, but the role of metal ions in modulating the timing and strength of such a suicidal response is underappreciated. Our growth-curve experiments consistently show that Mn^2+^-treated cultures outperform untreated controls under phage stress, confirming that pushing the infected subpopulation to die sooner pays dividends in population survival (Fig. 7B).

Beyond natural ecology, these findings have practical significance. Phage contamination remains a costly threat in industrial fermentations and probiotic manufacturing. Strategically adding Mn^2+^ or engineering bacterial strains with enhanced Mn^2+^ uptake could bolster CBASS function, preventing phage-driven batch failure. Alternatively, in a therapeutic setting—where we might want to harness phages to kill pathogens—targeting or controlling Mn^2+^ flux might either reinforce or diminish CBASS, depending on whether we aim to preserve the beneficial bacteria or facilitate phage-based clearance of harmful strains. Such “metal-ion-guided” microbial management is an emerging concept at the intersection of synthetic biology, industrial microbiology, and infection control.

Mn^2+^ has a higher affinity with DncV compared with Mg^2+^ (Kd = 1.26 µM vs Kd = 25.86 µM, Fig. 4E), providing a necessary basis for subsequent functional and structural analyses. *In vitro* experiments showed that Mn^2+^ further boosts DncV catalytic efficiency, accelerating cGAMP synthesis (Fig. 3). Quantum chemistry calculations and molecular docking revealed that Mn^2+^ forms shorter, stronger coordination bonds with the DncV than Mg^2+^, mirroring the high affinity measured by ITC (Fig. 4C and D). The tighter GTP binding with Mn^2+^ (Kd = 2.63 µM; Fig. S8C) arises, in turn, from reduced metal-ligand distances (Fig. 4D), supporting a mechanism where Mn^2+^ optimizes substrate interactions for accelerated catalysis. Thus, even in Mg^2+^-rich cells, infection-induced Mn^2+^ surges rapidly displace Mg^2+^ from DncV, hyper-activating the CBASS pathway and furnishing bacteria with a swift antiviral switch (Fig. 8). A surprising finding we also uncovered is that while folate-like molecules (e.g., tetrahydrofolate, THF) can significantly impede DncV-mediated cGAMP production, the addition of Mn^2+^ abrogates most of this folate-based inhibition (Fig. 5A and B), likely because Mn^2+^ reconfigures the cyclase active site in a way that folate cannot easily bind or block. To address this, we have performed additional ITC experiments and computational structural analyses. The ITC results showed that Mn^2+^-pretreated DncV exhibited a markedly reduced affinity for THF, whereas Mg^2+^-pretreatment had minimal effect (Fig. 5C, middle and right panels). This suggests that Mn^2+^ occupancy in the active site hinders THF binding, thereby alleviating inhibition. In addition, the docking revealed that THF binds to overlapping sites in both DncV-Mg^2+^-THF and DncV-Mn^2+^-THF complexes, primarily involving key residues in the active site (Fig. 5D). However, the number of hydrogen bonds differed: eight in the Mg^2+^ complex (Fig. 5D, left) versus seven in the Mn^2+^ complex (Fig. 5D, right). This reduction in stabilizing interactions under Mn^2+^ conditions likely contributes to weaker THF binding and thus diminished inhibition, as fewer hydrogen bonds correlate with lower affinity and easier dissociation of the inhibitor. These new findings provide a mechanistic explanation: Mn^2+^ induces subtle conformational changes or tighter metal-ligand coordination (as supported by our earlier binding energy calculations in Fig. 4C and D; Fig. S8A), which sterically or energetically disfavor THF binding without altering the overall site. From a therapeutic standpoint, if folate analogs are used to suppress bacterial growth or disrupt metabolism, bacteria might offset these effects by harnessing Mn^2+^ (45, 46). Conversely, if controlling Mn^2+^ is feasible, one might amplify the antibiotic efficacy of folate analogs by preventing the bacterium from rescuing cGAMP production.

While we have gained considerable insight into how Mn^2+^ galvanizes bacterial CBASS, several critical questions remain to be addressed. Although our computational energy calculations (Fig. 4C and Table 1) highlight Mn^2+^’s stronger binding interactions, crystal or cryo-EM structures of DncV in complex with Mn^2+^ would reveal precisely how the metal center is oriented. Parallel structural data in eukaryotic cGAS or insect cGLRs with Mn^2+^ bound would help confirm whether the active site rearrangements are truly homologous or represent convergent adaptation. The pathways that sense infection and signal for Mn^2+^ import are still murky. Do bacteria upregulate these transporters via classical stress responses (Our experimental results preliminarily show that phage infection elevates intracellular ROS levels in bacteria), or is there a specialized sensor that detects phage components (DNA, RNA, tail proteins, etc.)? Understanding the regulatory logic that couples phage detection to ion gating will shed light on how swiftly the cell can mount its cGAS-like response. Just as viruses in eukaryotes sometimes encode proteins that degrade or sequester cGAMP, phages might carry genes to sequester Mn^2+^ or sabotage DncV. Investigating whether phage genomes contain anti-CBASS modules—for example, proteins that block ion transport, degrade cGAMP, or sequester Mn^2+^—will be crucial for painting a full picture of the arms race.

Together, our study reveals that Mn^2+^ is more than a passive cofactor: it is a powerful driver of cGAMP synthesis in bacterial CBASS and, by extension, a likely unifying theme across cGAS-STING-like systems in eukaryotes. By mobilizing Mn^2+^ upon phage infection, bacteria accelerate the production of cGAMP, activating effectors such as CapV to trigger rapid cell death and block viral replication. Intriguingly, mammalian cGAS also exhibits heightened sensitivity to Mn^2+^, suggesting that metal ions are a deeply conserved currency of innate immunity. These parallels enhance our appreciation for how fundamentally similar “danger-sensing” nucleotidyltransferases can be—even across domains separated by billions of years of evolution. They also highlight the evolutionary rationale for maintaining robust mechanisms of ion homeostasis during infection.

## MATERIALS AND METHODS

### Bacterial strains, phages and growth conditions

The bacterial strains, phages, and plasmids used in this study are listed in Table S1 to S3. *E. coli* strains and derivatives were grown in Luria-Bertani (LB: 1% tryptone, 0.5% yeast extract, and 1% NaCl) medium at 37°C. Antibiotic concentrations were as follows: chloramphenicol, 20 µg/mL, ampicillin, 100 µg/mL, and kanamycin, 50 µg/mL. Phages (P1 and T4) were propagated on *E. coli* K12-MG1655 in liquid culture, their titer was determined using the small drop plaque assay method, as previously described (47).

### Cloning of the CBASS into *E. coli* K12-MG1655

The CBASS from *V. cholerae* El Tor N16961 was identified as locus tags VC0178–VC0181 (CBASS*^Vc^*), and the operon was taken together with its upstream and downstream intergenic regions, spanning the nucleotide range 178,424–183,957 in GenBank accession NC_002505. This construct was commercially synthesized and cloned by Sangon Biotech directly into the plasmid pBBR1MCS-2. The recombinant plasmid was transformed into *E. coli* K12-MG1655 by electroporation to obtain a strain containing the CBASS system. To generate point mutation plasmids, using plasmid pBBR1MCS-2-CBASS*^Vc^* as a template, site-directed mutagenesis was used to construct point mutation plasmids (QuickMutation Site-Directed Mutagenesis Kit, Cat# D0206M, Beyotime). The point mutation plasmids (containing pBBR1MCS-2-*cbass^Vc^*^D131A/D133A^, pBBR1MCS-2-*cbass^Vc^*^S62A^, and pBBR1MCS-2-*cbass^Vc^*^S62A/D131A/D133A^) were verified by DNA sequencing and transformed into K12-MG1655 by electroporation. The K12-MG1655 containing an empty pBBR1MCS-2 plasmid was used as a negative control.

### Plasmid construction

Primers used in this study are listed in Table S4. To express His_6_-tagged DncV and CapV, primers *dncV* BamH I F/*dncV* XhoI R and *capV* BamH I F/*capV* XhoI R were used to amplify *dncV* and *capV* fragments from the genome of *V. cholerae* (plasmid pBBR1MCS-2-CBASS*^Vc^* was used as the template DNA). The PCR products of *dncV* and *capV* were digested with BamHI and XhoI, respectively, and then inserted into vector pET21a to generate pET21a-*dncV* and pET21a-*capV*. To generate point mutation plasmids, using plasmids pET21a-*dncV* and pET21a-*capV* as templates, site-directed mutagenesis was used to construct point mutation plasmids. The point mutation plasmids pET21a-*dncV*^D131A/D133A^ and pET21a-*capV*^S62A^ were verified by DNA sequencing. The construction method of the His_6_-tagged DncE expression vector pET21a-*dncE* is the same as above. The plasmid sequences of all the recombinant plasmids used in this study were verified by DNA sequencing.

The plasmid pTargetF1-Δ*mntH*, pTargetF1-Δ*mntR,* and pTargetF1-Δ*mntP* was used to produce the Δ*mntH*, Δ*mntR,* and Δ*mntP* in-frame deletion mutants of *E. coli* K12-MG1655, respectively. Take the plasmid pTargetF1-Δ*mntP* construction as an example, the sequence of *mntP*-sg20 was amplified using the primers D-*mntP*-sg20-speI F/D-*mntP*-sg20 R, the *mntP* upstream fragment was amplified using the primers D-*mntP*-up F/^D-^*mntP* and the *mntP* downstream fragment was amplified using the primers. Next, the PCR fragments of *mntP*-sg20, *mntP* upstream, and *mntP* downstream were fused by overlapping PCR, and the PCR fragment was inserted into the vector pTargetF1 by Seamless Cloning (Seamless Cloning Kit, Cat# D7010S, Beyotime) to produce pTargetF1-Δ*mntP*. The construction method of the vector pTargetF1-Δ*mntH* and pTargetF1-Δ*mntR* is the same as above. The plasmid sequences of all the recombinant plasmids used in this study were verified by DNA sequencing.

### In-frame deletion in *E. coli* K12-MG1655

The deletion of *mntH*, *mntR,* and *mntP* in *E. coli* K12-MG1655 was performed using the CRISPR-Cas9 system (48). First, the plasmid pCas was transformed into *E. coli* K12-MG1655 by electroporation at 30°C. Then, the plasmid pTargetF1-Δ*mntH*, pTargetF1-Δ*mntR,* and pTargetF1-Δ*mntP* was also transformed into *E. coli* K12-MG1655 containing pCas in which arabinose (10 mM final concentration) was added for induction of λ-Red recombinase. The cells were recovered at 30°C for 2 h and spread onto LB agar containing chloramphenicol (Cm) and kanamycin (Km). *E. coli* K12-MG1655 mutants with deletion of the *mntH*, *mntR,* and *mntP* gene were identified by PCR and DNA sequencing, and then the pTargetF1 derivative and pCas in the Δ*mntH*, Δ*mntR,* and Δ*mntP* mutants were successively eliminated by β-D-1-thiogalactopyranoside (IPTG) (Cat# HY-15921, CAS: 367-93-1, MCE) induction and overnight incubation at 37°C, respectively.

### Determination of metal ion concentration *in vivo*

Bacterial intracellular ion content was determined as described previously, with slight modifications (18, 49). All samples were analyzed by ICP-MS (Varian 802 MS), and the results were corrected using the appropriate buffers for reference and dilution factors. The strains were grown at 37°C with 5 mL LB liquid medium until the stationary phase, and these strains were collected and rinsed twice with M9 (6 g/L Na_2_HPO4, 3 g/L KH_2_PO_4_, 1 g/L NH_4_Cl, 0.5 g/L NaCl, 2 mM MgSO_4_, 0.1 mM CaCl_2_, and 20 mM Glucose) liquid medium. Then, they were diluted 1:100 in 50 ml M9 liquid medium and incubated until OD_600_ = 1.0. The above bacterial culture was centrifuged at 4,500 rpm for 20 min to collect bacterial precipitation and washed twice with phosphate-buffered saline (PBS; 137 mM NaCl, 2.67 mM KCl, 10 mM Na_2_HPO_4_, 2 mM KH_2_PO_4_, Cat# 41403ES76, YEASEN). The bacterial pellet was resuspended in BugBuster Protein Extraction Reagent (Cat# 71456, Novagen) and left to spin overnight at 4°C. The samples were centrifuged at 12,000 rpm for 20 min, and the protein concentration in the supernatant was determined. The supernatant was diluted in 2% nitric acid (chromatographic grade) according to 1:100 and spun for 12 h. Centrifuge again and detect the metal ion concentration in the supernatant using ICP-MS. Similarly, the bacteria were infected with phage for 10 min and then collected, and the intracellular metal ion concentration was determined by ICP-MS.

### RNA extraction and quantitative real-time PCR

Bacteria were infected with phage for 10 min and then collected, and total RNA was purified by the total RNA isolation kit (Cat# 19301ES50, YEASEN) according to the manufacturer’s protocol. The purity and concentration of the sample RNA were determined, and the cDNA was synthesized from the RNA template by using cDNA Synthesis SuperMix (EasyScript One-Step gDNA Removal and cDNA Synthesis SuperMix, Cat# AE311 TransGen Biotech, Beijing, China). Quantitative real-time PCR (qRT-PCR) was used to quantify gene expression of *mntH*. qRT-PCR was performed in a CFX96 real-time PCR detection system (BioRad, USA) with TransStart green qPCR SuperMix (PerfectStart Green qPCR SuperMix, Cat# AQ601, TransGen Biotech, Beijing, China). The qRT-PCR primers are listed in Table S4, and the qRT-PCR parameters were 95°C for 150 s followed by 40 cycles of 95°C for 10 s and 58°C for 30 s, from 65°C to 95°C for 5 s, and 37°C for 30 s. The relative abundance of 16S rRNA was used as the internal standard for standardization of results.

### ROS assay

The bacteria were collected during the late logarithmic growth phase and washed twice with PBS for the ROS experiment. The phage was added to a multiplicity of infection (MOI, ratio of phages to bacteria) of 0.1 for 10 or 40 min. Then, the bacterial pellet was harvested by centrifugation at 4,500 rpm for 5 min. The bacterial pellets were resuspended with 1 mL H_2_DCFDA dye (10 µM solution) (Cat# 50101ES01, CAS: 4091-99-0, YEASEN) and incubated for 30 min in the dark. Lastly, the bacterial pellets were cleaned with PBS buffer two times to remove unbound dye and resuspended again with 200 µL PBS. The fluorescence signals (H_2_DCFDA) were measured using a SpectraMax M2 Plate Reader (Molecular Devices) with excitation/emission wavelengths of 488/525 nm.

### Protein structure analysis, expression, and purification

The plasmids pET21a-*dncV*, pET21a-*dncV^D131A/D133A^*, pET21a-*capV*, and pET21a-*capV^S62A^* were transformed into *E. coli* BL21 (DE3) to express protein DncV, DncV^D131A/D133A^, CapV, and CapV^S62A^. These expressing strains were grown overnight on a 37°C shaker with 220 rpm. Next, these strains were diluted 1:100 in LB liquid medium and incubated until OD_600_ = 0.4. 0.3 mM IPTG was added to the LB liquid medium and cultivation was continued for 12 h at 20°C shaker at 160 rpm. Harvested cells were washed twice and resuspended with His binding buffer (50 mM Tris-HCl, 150 mM NaCl, 10 mM imidazole, and pH = 8.0). The cells were further lysed by sonication. Then, protein DncV, DncV^D131A/D133A^, CapV, and CapV^S62A^ were purified in His·Bind resin (Cat# 70666, Novagen, Madison, WI), and eluted His-tagged protein with elution buffer (50 mM Tris-HCl, 150 mM NaCl, 250 mM imidazole, and pH = 8.0). Lastly, protein was dialyzed into buffer (50 mM Tris-HCl, 150 mM NaCl, 10% [vol/vol] glycerol, pH = 8.0) at 4°C overnight, respectively, and the proteins were aliquoted (200 µL per tube) and flash-frozen with liquid nitrogen for conservation at −80°C until used.

### *In vitro* enzyme activity of DncV assays

The enzyme activity of protein DncV was detected as previously described (32, 33, 50). 2 µM protein DncV was added into the reaction system (50 mM Tris-HCl, pH 7.5, 1 mM metal ions, 100 µM ATP, and 100 µM GTP) for 30 min at 37°C. Next, all reaction mixtures were heated for 10 min at 98°C and centrifuged for 10 min at 12,000 rpm to obtain supernatant. Lastly, all samples were detected by HPLC (Waters e2695 Separations Module) with C18 Symmetry Shield RP18 5 µm (4.6 × 250 mm) (Cat# 186000109, purchased from Waters Corporation, Milford, MA, USA) in 30°C. The wavelength of the UV detector (Waters 2998 PDA Detector) was 254 nm. The supernatant was eluted with 98% Na_2_HPO_4_ (pH 5.2, 150 mM) and 2% acetonitrile (chromatography grade, purchased from Merck, Darmstadt, Germany) in 30 min and the flow rate was 1 ml/min. The data analysis was performed using Empower software (Waters). The above reaction system is the basic reaction system, and subsequent experiments need to be adjusted according to the purpose, including the type of protein in the reaction system, the amount of protein added, the type of metal ion, the type of reaction substrate, and the reaction time. All metal ion reagents were purchased from Aladdin, and detailed information can be found in the Supplementary Information. ATP, GTP, c-di-AMP, c-di-GMP, and 3′3′-cGAMP were purchased from Sigma; detailed information can be found in the Supplementary Information. The detailed information of reagents and resources was provided in the Supplementary Information Table S5.

### Isothermal titration calorimetry

The ITC experiment was carried out using a Nano ITC Low Volume isothermal calorimeter (TA Instruments, New Castle, DE). The ITC instrument was controlled by the ITCRun software, and all data analysis was performed using Nano Analyze software (TA Instruments) with an independent binding model. For titrations with metal ions (Mg^2+^ and Mn^2+^), the protein DncV or DncE was dialyzed against Tris-HCl buffer (50 mM Tris-HCl, 150 mM NaCl, and pH = 8.0) and diluted to 25 µM, while Mg^2+^ or Mn^2+^ was diluted with the same buffer to 500 µM. All proteins and metal solutions were degassed for 25 min prior to titration. 400 µL protein and 50 µL metal solution were added to the sample cell and syringe, respectively. After a stable baseline had been achieved, the ligand solution was added with a total of 25 injections of 2 µL into the protein solution. The stirring speed was 200 rpm at 25°C for all ITC experiments. The control experiment in the absence of protein was performed to measure the heat generated due to metal solution dilution in the Tris buffer. Specifically, blank titrations of the metal solution into the dialysis buffer were performed to correct for the dilution heat of the metal solution. To test whether Mn^2+^ affects protein binding to Mg^2+^, 25 µM protein was incubated with 10 µM Mn^2+^ at 4°C for 30 min, and the affinity of protein-Mn^2+^ and Mg^2+^ was measured by ITC experiment. Similarly, to test whether Mg^2+^ affects protein binding to Mn^2+^, 25 μM protein was incubated with 10 µM Mg^2+^ at 4°C for 30 min, and the affinity of protein-Mg^2+^ and Mn^2+^ was measured by ITC experiment. For titrations with tetrahydrofolate (THF) (Cat# T3125, CAS: 135-16-0, Sigma-Aldrich), the protein DncV was dialyzed against Tris-HCl buffer and diluted to 30 µM, while THF was diluted with the same buffer to 600 µM. The remaining experimental procedures were the same as above in the ITC assay. To test whether Mg^2+^ or Mn^2+^ affects protein binding to THF, 30 µM protein was incubated with 10 µM Mg^2+^ or Mn^2+^ at 4°C for 30 min, and the affinity of protein-Mg^2+^ or Mn^2+^ and THF was measured by ITC experiment, respectively. For titrations with ATP and GTP, the protein DncV was dialyzed against Tris-HCl buffer and diluted to 10 µM, while THF was diluted with the same buffer to 150 µM. The remaining experimental procedures were the same as above in the ITC assay. To test whether Mg^2+^ or Mn^2+^ affects protein binding to ATP or GTP, 10 µM protein was incubated with 10 µM Mg^2+^ or Mn^2+^ at 4°C for 30 min, and the affinity of protein-Mg^2+^ or Mn^2+^ and ATP or GTP was measured by ITC experiment, respectively.

### Quantum chemistry methods

The affinity of protein DncV for Mg^2+^ and Mn^2+^ was studied by using quantum chemical methods, and this part of the experiment was analyzed by Sichuan Model Technology Co., Ltd. (https://www.modekeji.cn/). The initial structure of protein DncV was extracted from the crystal structure of the protein (PDB ID: 4U03), retaining GTP as well as amino acid residues coordinated to metal ions (Asp131, Asp133, Arg182, and Asp193), and amino acids were capped at the N- and C-termini using acetyl and hydrogen atoms, respectively. The system was constructed by replacing Mg^2+^ with Mn^2+^ to obtain Mn^2+^ bound to GTP. The two systems for Mg^2+^ and Mn^2+^ were optimized at the PBE0-D3/def2-SVP level (51–53), with the aqueous solvent environment taken into account in the calculations. The optimized structures were used to calculate the energies at the PBE0-D3/def2-TZVP level (54), and the Basis Set Superposition Error (BSSE) was added to the calculations to be more positive (55, 56). The final binding energy was calculated using the following equation:


$$E_{\text{interaction}}=E_{\text{complex}}-E_{\text{protein}}-E_{\left(\text{Mg/Mn}\right)}+E_{\text{BSSE}}$$

Specifically, *E*_interaction_ is the energy of the protein and metal ions, *E*_complex_ is the energy of the complex, *E*_protein_ is the energy of the protein DncV amino acid residues, *E*_(Mg/Mn)_ is the energy of the Mg^2+^ or Mn^2+^, and the *E*_BSSE_ denotes the more positive energy of BSSE.

### Molecular docking

Protein models of DncV were retrieved from the Protein Data Bank (PDB) (https://www.rcsb.org/), while the structure file of ligands was sourced from the PubChem database (https://pubchem.ncbi.nlm.nih.gov/). For the molecular docking model of protein DncV with metal ions, first obtain the structures of the protein (PDB ID: 4U03) and the metal ions (Mg^2+^: 5462224, Mn^2+^: 23930, from PubChem database), respectively. Then perform the docking calculations with AutoDock Vina, retain the complex conformation with the lowest binding energy, and save it as a PDB file. Finally, visualize the results in PyMOL (*PyMOL |* pymol.org) and export images displaying molecular interactions such as hydrogen bonds and hydrophobic contacts (57). To construct the ternary docking model of DncV protein, metal ions (Mg^2+^ or Mn^2+^), and THF (135403828, from PubChem database), first retrieve the structures of all three components, perform precise docking with AutoDock Vina, retain the energetically optimal conformation, and output it as a PDB file; subsequently, render it in PyMOL to export a clear 3D map highlighting hydrogen bonds, hydrophobic contacts, and other relevant interactions.

### Fluorogenic biochemical assay for CapV activity

The enzyme activity of phospholipase CapV was assayed by using Resorufin butyrate (Cat# sc-208302, CAS: 15585-42-9, Santa Cruz Biotechnology) as substrate. The protein CapV was diluted in buffer (50 mM Na_3_PO4, pH 7.4, 300 mM NaCl, and 10% glycerol) to a final concentration of 1.77 µM. The Resorufin butyrate was dissolved in DMSO (Cat# HY-Y0320C, MCE) and further diluted in buffer (50 mM Na_3_PO4, pH 7.4, 300 mM NaCl, and 10% glycerol) to a final concentration of 64 µM. The diluted protein CapV was mixed with the diluted substrate in a final assay volume of 50 µL and added to a 96-well plate all-black plate (Cat# FCP966, Beyotime), and the phospholipase enzyme activity was determined by detecting fluorescence using a Microplate Reader (SpectraMax M2, Molecular Devices). Phospholipase enzyme activity was assayed at a constant temperature of 37°C with an excitation wavelength of 550 nm and an emission wavelength of 591 nm, once every 30 s for 10 min. A regression was fitted to the output values for each reaction over time, and the slope of this linear regression fit was used to determine the initial reaction rate (FU s^−1^). Data represent mean ± SEM from three biological replicates.

### Bacterial survival assay

*E. coli* K12-MG1655 containing the CBASS system was grown in LB liquid medium overnight at 37°C, and the strain was diluted 1:100 in LB liquid medium until the early phase (OD_600_ = 0.6). *E. coli* K12-MG1655 containing the CBASS system (no Mn^2+^/Mn^2+^) was infected with phage P1 (MOI = 1) and then collected at specific time points (0 min, 40 min, or 80 min). The samples were centrifuged at 12,000 rpm for 3 min. The resulting pellet was resuspended in 500 µL propidium iodide (PI) solution (Beyotime, ST511), filtered through a 300-mesh sieve, and analyzed by flow cytometry (CytoFLEX LX, BECKMAN COULTER). Gating was performed sequentially in CytExpert: debris was first excluded on an FSC-A/SSC-A plot, doublets were then removed by FSC-H/FSC-A, and membrane-compromised (non-viable) bacteria were identified with a PI fluorescence gate; 10,000 events were recorded per sample, and all fluorescence intensity data were processed within the same software. Fluorescence intensity data were processed using CytExpert software to quantify bacterial survival based on membrane integrity.

### Phage-infection dynamics in liquid medium

*E. coli* K12-MG1655 containing the CBASS system was grown in LB liquid medium overnight at 37°C, and the strain was diluted 1:100 in LB liquid medium until the early phase (OD_600_ = 0.3). The strains (no Mn^2+^/Mn^2+^) were mixed with phage P1 (MOI = 0, 0.1, and 1). The mixture was vortexed and mixed to aspirate 180 µL and then added to a 96-well plate, and the OD_600_ absorbance value of the bacteria was detected every 5 min at 37°C, 180 rpm. The growth curve was plotted according to the absorbance value with Graph Prism 9.

### Plaque assays

Strains were grown on a shaker at 37°C, 220 rpm, then they were diluted 1:100 in LB liquid medium and incubated until OD_600_ = 0.3. 300 µL of diluted phage culture was added to 300 µL of the different bacterial strains in 1.5 mL EP tube to achieve a multiplicity of infection of 0.01. The mixture was harvested by centrifugation at 4°C, 12,000 rpm for 3 min, and the lysate was filtered by a 0.22 µm filter. 200 µL of *E. coli* K12-MG1655 (OD_600_ = 1.0) was added into 5 ml LB of 0.5% agar (top agar) and poured over resolidified 1.8% LB bottom agar. A 10-fold serial dilution of the lysate in LB liquid medium was spotted onto them, let it stand at room temperature for 30 min before moving to the incubator, and growing at 37°C for 4–8 h. Plaques were counted and analyzed to determine the defense of different strains against phages.

### Statistical analysis

Statistical analyses were performed using GraphPad Prism Software 9. *P* values were analyzed using unpaired, two-tailed Student’s t-test, one-way or two-way ANOVA test. Error bars indicate ± SEM. Statistical significance is denoted in figures by asterisks. *P < 0.05; **P < 0.01; ***P < 0.001, ****P < 0.0001.

## SUPPLEMENTAL MATERIAL

Supplemental material mbio.02758-25-s0001.docx Fig. S1 to S13 and Table S1 to S5.

ASM does not own the copyrights to Supplemental Material that may be linked to, or accessed through, an article. The authors have granted ASM a non-exclusive, world-wide license to publish the Supplemental Material files. Please contact the corresponding author directly for reuse.

## References

[1] Whiteley AT, Eaglesham JB, de Oliveira Mann CC, Morehouse BR, Lowey B, Nieminen EA, Danilchanka O, King DS, Lee ASY, Mekalanos JJ, Kranzusch PJ. 2019. Bacterial cGAS-like enzymes synthesize diverse nucleotide signals. Nature 567:194–199. doi:10.1038/s41586-019-0953-5 30787435 PMC6544370

[2] Bernheim A, Sorek R. 2020. The pan-immune system of bacteria: antiviral defence as a community resource. Nat Rev Microbiol 18:113–119. doi:10.1038/s41579-019-0278-2 31695182

[3] Duncan-Lowey B, Kranzusch PJ. 2022. CBASS phage defense and evolution of antiviral nucleotide signaling. Curr Opin Immunol 74:156–163. doi:10.1016/j.coi.2022.01.002 35123147

[4] Lowey B, Whiteley AT, Keszei AFA, Morehouse BR, Mathews IT, Antine SP, Cabrera VJ, Kashin D, Niemann P, Jain M, Schwede F, Mekalanos JJ, Shao S, Lee ASY, Kranzusch PJ. 2020. CBASS immunity uses CARF-related effectors to sense 3’-5’- and 2’-5’-linked cyclic oligonucleotide signals and protect bacteria from phage infection. Cell 182:38–49. doi:10.1016/j.cell.2020.05.019 32544385 PMC7728545

[5] Hobbs SJ, Kranzusch PJ. 2024. Nucleotide immune signaling in CBASS, pycsar, thoeris, and CRISPR antiphage defense. Annu Rev Microbiol 78:255–276. doi:10.1146/annurev-micro-041222-024843 39083849 PMC12335278

[6] Tak U, Walth P, Whiteley AT. 2023. Bacterial cGAS-like enzymes produce 2’,3’-cGAMP to activate an ion channel that restricts phage replication. bioRxiv:2023.07.24.550367. doi:10.1101/2023.07.24.550367

[7] Slavik KM, Kranzusch PJ. 2023. CBASS to cGAS-STING: the origins and mechanisms of nucleotide second messenger immune signaling. Annu Rev Virol 10:423–453. doi:10.1146/annurev-virology-111821-115636 37380187 PMC12337107

[8] Millman A, Melamed S, Amitai G, Sorek R. 2020. Diversity and classification of cyclic-oligonucleotide-based anti-phage signalling systems. Nat Microbiol 5:1608–1615. doi:10.1038/s41564-020-0777-y 32839535 PMC7610970

[9] Kranzusch PJ, Lee ASY, Wilson SC, Solovykh MS, Vance RE, Berger JM, Doudna JA. 2014. Structure-guided reprogramming of human cGAS dinucleotide linkage specificity. Cell 158:1011–1021. doi:10.1016/j.cell.2014.07.028 25131990 PMC4157622

[10] Kato K, Ishii R, Hirano S, Ishitani R, Nureki O. 2015. Structural basis for the catalytic mechanism of DncV, bacterial homolog of cyclic GMP-AMP synthase. Structure 23:843–850. doi:10.1016/j.str.2015.01.023 25865248

[11] Hopfner KP, Hornung V. 2020. Molecular mechanisms and cellular functions of cGAS-STING signalling. Nat Rev Mol Cell Biol 21:501–521. doi:10.1038/s41580-020-0244-x 32424334

[12] Wang C, Sun Z, Zhao C, Zhang Z, Wang H, Liu Y, Guo Y, Zhang B, Gu L, Yu Y, Hu Y, Wu J. 2021. Maintaining manganese in tumor to activate cGAS-STING pathway evokes a robust abscopal anti-tumor effect. J Control Release 331:480–490. doi:10.1016/j.jconrel.2021.01.036 33545219

[13] Burdette DL, Monroe KM, Sotelo-Troha K, Iwig JS, Eckert B, Hyodo M, Hayakawa Y, Vance RE. 2011. STING is a direct innate immune sensor of cyclic di-GMP. Nature 478:515–518. doi:10.1038/nature10429 21947006 PMC3203314

[14] Gui X, Yang H, Li T, Tan X, Shi P, Li M, Du F, Chen ZJ. 2019. Autophagy induction via STING trafficking is a primordial function of the cGAS pathway. Nature 567:262–266. doi:10.1038/s41586-019-1006-9 30842662 PMC9417302

[15] Bhojwani-Cabrera AM, Bautista-Garcia A, Neubrand VE, Membrive-Jimenez FA, Bramini M, Martin-Oliva D, Cuadros MA, Marin-Teva JL, Navascues J, Vangheluwe P, Sepulveda MR. 2024. Upregulation of the secretory pathway Ca(2+)/Mn(2+)-ATPase isoform 1 in LPS-stimulated microglia and its involvement in Mn(2+)-induced Golgi fragmentation. Glia 72:1201–1214. doi:10.1002/glia.24528 38482950

[16] Song Y, Liu Y, Teo HY, Hanafi ZB, Mei Y, Zhu Y, Chua YL, Lv M, Jiang Z, Liu H. 2021. Manganese enhances the antitumor function of CD8^+^ T cells by inducing type I interferon production. Cell Mol Immunol 18:1571–1574. doi:10.1038/s41423-020-00524-4 32801367 PMC8166851

[17] Zhang R, Wang C, Guan Y, Wei X, Sha M, Yi M, Jing M, Lv M, Guo W, Xu J, Wan Y, Jia XM, Jiang Z. 2021. Manganese salts function as potent adjuvants. Cell Mol Immunol 18:1222–1234. doi:10.1038/s41423-021-00669-w 33767434 PMC8093200

[18] Zhu L, Xu L, Wang C, Li C, Li M, Liu Q, Wang X, Yang W, Pan D, Hu L, Yang Y, Lu Z, Wang Y, Zhou D, Jiang Z, Shen X. 2021. T6SS translocates a micropeptide to suppress STING-mediated innate immunity by sequestering manganese. Proc Natl Acad Sci USA 118:e2103526118. doi:10.1073/pnas.2103526118 34625471 PMC8545469

[19] Wang C, Guan Y, Lv M, Zhang R, Guo Z, Wei X, Du X, Yang J, Li T, Wan Y, Su X, Huang X, Jiang Z. 2018. Manganese increases the sensitivity of the cGAS-STING pathway for double-stranded DNA and is required for the host defense against DNA viruses. Immunity 48:675–687. doi:10.1016/j.immuni.2018.03.017 29653696

[20] Zhao Z, Ma Z, Wang B, Guan Y, Su XD, Jiang Z. 2020. Mn2+ directly activates cGAS and structural analysis suggests Mn2+ induces a noncanonical catalytic synthesis of 2′3′-cGAMP. Cell Rep 32:108053. doi:10.1016/j.celrep.2020.108053 32814054

[21] Papp-Wallace KM, Maguire ME. 2006. Manganese transport and the role of manganese in virulence. Annu Rev Microbiol 60:187–209. doi:10.1146/annurev.micro.60.080805.142149 16704341

[22] Aguirre JD, Culotta VC. 2012. Battles with iron: manganese in oxidative stress protection. J Biol Chem 287:13541–13548. doi:10.1074/jbc.R111.312181 22247543 PMC3340200

[23] Lisher JP, Giedroc DP. 2013. Manganese acquisition and homeostasis at the host-pathogen interface. Front Cell Infect Microbiol 3:91. doi:10.3389/fcimb.2013.00091 24367765 PMC3851752

[24] Kehres DG, Janakiraman A, Slauch JM, Maguire ME. 2002. SitABCD is the alkaline Mn(2+) transporter of Salmonella enterica serovar Typhimurium. J Bacteriol 184:3159–3166. doi:10.1128/JB.184.12.3159-3166.2002 12029031 PMC135093

[25] Obeng SK, Kulhánek M, Balík J, Černý J, Sedlář O. 2024. Manganese: from soil to human health-A comprehensive overview of its biological and environmental significance. Nutrients 16:3455. doi:10.3390/nu16203455 39458451 PMC11510450

[26] Si M, Zhao C, Burkinshaw B, Zhang B, Wei D, Wang Y, Dong TG, Shen X. 2017. Manganese scavenging and oxidative stress response mediated by type VI secretion system in Burkholderia thailandensis Proc Natl Acad Sci USA 114:E2233–E2242. doi:10.1073/pnas.1614902114 28242693 PMC5358365

[27] Bosma EF, Rau MH, van Gijtenbeek LA, Siedler S. 2021. Regulation and distinct physiological roles of manganese in bacteria. FEMS Microbiol Rev 45:fuab028. doi:10.1093/femsre/fuab028 34037759 PMC8632737

[28] Colomer-Winter C, Flores-Mireles AL, Baker SP, Frank KL, Lynch AJL, Hultgren SJ, Kitten T, Lemos JA. 2018. Manganese acquisition is essential for virulence of Enterococcus faecalis. PLoS Pathog 14:e1007102. doi:10.1371/journal.ppat.1007102 30235334 PMC6147510

[29] Wu J, McAuliffe O, O’Byrne CP. 2023. Manganese uptake mediated by the NRAMP-type transporter MntH is required for acid tolerance in Listeria monocytogenes. Int J Food Microbiol 399:110238. doi:10.1016/j.ijfoodmicro.2023.110238 37148667

[30] Tsolis RM, Bäumler AJ, Heffron F. 1995. Role of Salmonella typhimurium Mn-superoxide dismutase (SodA) in protection against early killing by J774 macrophages. Infect Immun 63:1739–1744. doi:10.1128/iai.63.5.1739-1744.1995 7729880 PMC173218

[31] Barnese K, Gralla EB, Valentine JS, Cabelli DE. 2012. Biologically relevant mechanism for catalytic superoxide removal by simple manganese compounds. Proc Natl Acad Sci USA 109:6892–6897. doi:10.1073/pnas.1203051109 22505740 PMC3344976

[32] Davies BW, Bogard RW, Young TS, Mekalanos JJ. 2012. Coordinated regulation of accessory genetic elements produces cyclic di-nucleotides for V. cholerae virulence. Cell 149:358–370. doi:10.1016/j.cell.2012.01.053 22500802 PMC3620040

[33] Hooy RM, Massaccesi G, Rousseau KE, Chattergoon MA, Sohn J. 2020. Allosteric coupling between Mn2+ and dsDNA controls the catalytic efficiency and fidelity of cGAS. Nucleic Acids Res 48:4435–4447. doi:10.1093/nar/gkaa084 32170294 PMC7192592

[34] Radzikowska U, Eljaszewicz A, Tan G, Stocker N, Heider A, Westermann P, Steiner S, Dreher A, Wawrzyniak P, Rückert B, Rodriguez-Coira J, Zhakparov D, Huang M, Jakiela B, Sanak M, Moniuszko M, O’Mahony L, Jutel M, Kebadze T, Jackson DJ, Edwards MR, Thiel V, Johnston SL, Akdis CA, Sokolowska M. 2023. Rhinovirus-induced epithelial RIG-I inflammasome suppresses antiviral immunity and promotes inflammation in asthma and COVID-19. Nat Commun 14:2329. doi:10.1038/s41467-023-37470-4 37087523 PMC10122208

[35] Traut TW. 1994. Physiological concentrations of purines and pyrimidines. Mol Cell Biochem 140:1–22. doi:10.1007/BF00928361 7877593

[36] Zhu D, Wang L, Shang G, Liu X, Zhu J, Lu D, Wang L, Kan B, Zhang J, Xiang Y. 2014. Structural biochemistry of a vibrio cholerae dinucleotide cyclase reveals cyclase activity regulation by folates. Mol Cell 55:931–937. doi:10.1016/j.molcel.2014.08.001 25201413

[37] Cohen D, Melamed S, Millman A, Shulman G, Oppenheimer-Shaanan Y, Kacen A, Doron S, Amitai G, Sorek R. 2019. Cyclic GMP-AMP signalling protects bacteria against viral infection. Nature 574:691–695. doi:10.1038/s41586-019-1605-5 31533127

[38] Lopatina A, Tal N, Sorek R. 2020. Abortive infection: bacterial suicide as an antiviral immune strategy. Annu Rev Virol 7:371–384. doi:10.1146/annurev-virology-011620-040628 32559405

[39] Lv M, Chen M, Zhang R, Zhang W, Wang C, Zhang Y, Wei X, Guan Y, Liu J, Feng K, Jing M, Wang X, Liu YC, Mei Q, Han W, Jiang Z. 2020. Manganese is critical for antitumor immune responses via cGAS-STING and improves the efficacy of clinical immunotherapy. Cell Res 30:966–979. doi:10.1038/s41422-020-00395-4 32839553 PMC7785004

[40] Jenson JM, Chen ZJ. 2024. cGAS goes viral: a conserved immune defense system from bacteria to humans. Mol Cell 84:120–130. doi:10.1016/j.molcel.2023.12.005 38181755 PMC11168419

[41] Wang C, Zhang R, Wei X, Lv M, Jiang Z. 2020. Metalloimmunology: the metal ion-controlled immunity. Adv Immunol 145:187–241. doi:10.1016/bs.ai.2019.11.007 32081198

[42] Holleufer A, Winther KG, Gad HH, Ai X, Chen Y, Li L, Wei Z, Deng H, Liu J, Frederiksen NA, Simonsen B, Andersen LL, Kleigrewe K, Dalskov L, Pichlmair A, Cai H, Imler JL, Hartmann R. 2021. Two cGAS-like receptors induce antiviral immunity in Drosophila. Nature 597:114–118. doi:10.1038/s41586-021-03800-z 34261128

[43] Zhang Y, Feng Y. 2025. A broad anti-anti-CRISPR strategy. Nat Chem Biol 21:312–313. doi:10.1038/s41589-024-01743-w 39406979

[44] Cao X, Xiao Y, Huiting E, Cao X, Li D, Ren J, Fedorova I, Wang H, Guan L, Wang Y, Li L, Bondy-Denomy J, Feng Y. 2024. Phage anti-CBASS protein simultaneously sequesters cyclic trinucleotides and dinucleotides. Mol Cell 84:375–385. doi:10.1016/j.molcel.2023.11.026 38103556 PMC11102597

[45] Brenzinger S, Airoldi M, Ogunleye AJ, Jugovic K, Amstalden MK, Brochado AR. 2024. The Vibrio cholerae CBASS phage defence system modulates resistance and killing by antifolate antibiotics. Nat Microbiol 9:251–262. doi:10.1038/s41564-023-01556-y 38172623

[46] Severin GB, Ramliden MS, Ford KC, Van Alst AJ, Sanath-Kumar R, Decker KA, Hsueh BY, Chen G, Yoon SH, Demey LM, O’Hara BJ, Rhoades CR, DiRita VJ, Ng W-L, Waters CM. 2023. Activation of a Vibrio cholerae CBASS anti-phage system by quorum sensing and folate depletion. mBio 14:e0087523. doi:10.1128/mbio.00875-23 37623317 PMC10653837

[47] Doron S, Melamed S, Ofir G, Leavitt A, Lopatina A, Keren M, Amitai G, Sorek R. 2018. Systematic discovery of antiphage defense systems in the microbial pangenome. Science 359:eaar4120. doi:10.1126/science.aar4120 29371424 PMC6387622

[48] Jiang Y, Chen B, Duan C, Sun B, Yang J, Yang S. 2015. Multigene editing in the Escherichia coli genome via the CRISPR-Cas9 system. Appl Environ Microbiol 81:2506–2514. doi:10.1128/AEM.04023-14 25636838 PMC4357945

[49] Puri S, Hohle TH, O’Brian MR. 2010. Control of bacterial iron homeostasis by manganese. Proc Natl Acad Sci USA 107:10691–10695. doi:10.1073/pnas.1002342107 20498065 PMC2890801

[50] Wang X, Hao X, Yang Y, Jia S, Chen Y, Yang W, Luo Y, Xie Z, Gu Y, Wu Y, Zhang F, Li M, Wang Y, Shen X, Xu L. 2024. A phosphodiesterase CpdB in Yersinia pseudotuberculosis degrades CDNs to inhibit innate immune response. Vet Microbiol 297:110194. doi:10.1016/j.vetmic.2024.110194 39084162

[51] Vázquez H. 2022. Toward density-functional theory-based structure-conductance relationships in single molecule junctions. J Phys Chem Lett 13:9326–9331. doi:10.1021/acs.jpclett.2c02349 36178209

[52] Grimme S, Antony J, Ehrlich S, Krieg H. 2010. A consistent and accurate ab initio parametrization of density functional dispersion correction (DFT-D) for the 94 elements H-Pu. J Chem Phys 132:154104. doi:10.1063/1.3382344 20423165

[53] Weigend F, Ahlrichs R. 2005. Balanced basis sets of split valence, triple zeta valence and quadruple zeta valence quality for H to Rn: design and assessment of accuracy. Phys Chem Chem Phys 7:3297–3305. doi:10.1039/b508541a 16240044

[54] Weigend F. 2006. Accurate Coulomb-fitting basis sets for H to Rn. Phys Chem Chem Phys 8:1057–1065. doi:10.1039/b515623h 16633586

[55] Mata RA, Werner HJ. 2006. Calculation of smooth potential energy surfaces using local electron correlation methods. J Chem Phys 125:184110. doi:10.1063/1.2364487 17115741

[56] Ribeiro J, Ríos-Vera C, Melo F, Schüller A. 2019. Calculation of accurate interatomic contact surface areas for the quantitative analysis of non-bonded molecular interactions. Bioinformatics 35:3499–3501. doi:10.1093/bioinformatics/btz062 30698657 PMC6748739

[57] Schiffrin B, Radford SE, Brockwell DJ, Calabrese AN. 2020. PyXlinkViewer: a flexible tool for visualization of protein chemical crosslinking data within the PyMOL molecular graphics system. Protein Sci 29:1851–1857. doi:10.1002/pro.3902 32557917 PMC7380677

