# Supplementary material for: Manganese activates the CBASS immunity to protect bacteria from phage infection
Source: mBio. 2025 Dec 8;17(1):e02758-25. doi: 10.1128/mbio.02758-25 (PMC12802263; doi:10.1128/mbio.02758-25)
Supplement: Supplemental material — Fig. S1 to S13 and Table S1 to S5. [file mbio.02758-25-s0001.docx]

**Supplemental Information**

**Manganese activates the CBASS immunity to protect bacteria from phage infection**

Xiao Wang^a#^, Yongdong Li^b#^, Xiao Wang^a#^, Wenjing Zhang^a^, Muohua Liu^a^ , Xinwei Hao^a^, Shukun Chen^a^, Tianyuan Chang^a^, Conghui Wu^a^, Chonghua Hao^c^, Li Song^d^, Hongxia Ni^b^, Yi Chen^b^*, Xihui Shen^a^*, Lei Xu^a^*

^a^State Key Laboratory for Crop Stress Resistance and High-Efficiency Production, Shaanxi Key Laboratory of Agricultural and Environmental Microbiology, College of Life Sciences, Northwest A&F University, Yangling, Shaanxi, 712100, China;

^b^Ningbo Municipal Center for Disease Control and Prevention, Ningbo, 315010, China;

^c^Shanxi Provincial People’s Hospital Affiliated to Shanxi Medical University, Taiyuan, 030000, China;

^d^State Key Laboratory for Crop Stress Resistance and High-Efficiency Production, Shaanxi Key Laboratory of Agricultural and Environmental Microbiology, College of Natural Resources and Environment, Northwest A&F University, Yangling, Shaanxi, 712100, China.

^#^These authors contributed equally to this work.

*For correspondence:

Lei Xu (xulei@nwafu.edu.cn)

Xihui Shen (xihuishen@nwsuaf.edu.cn)

Yi Chen ([30279068@qq.com](mailto:30279068@qq.com))

# Supplementary Figures


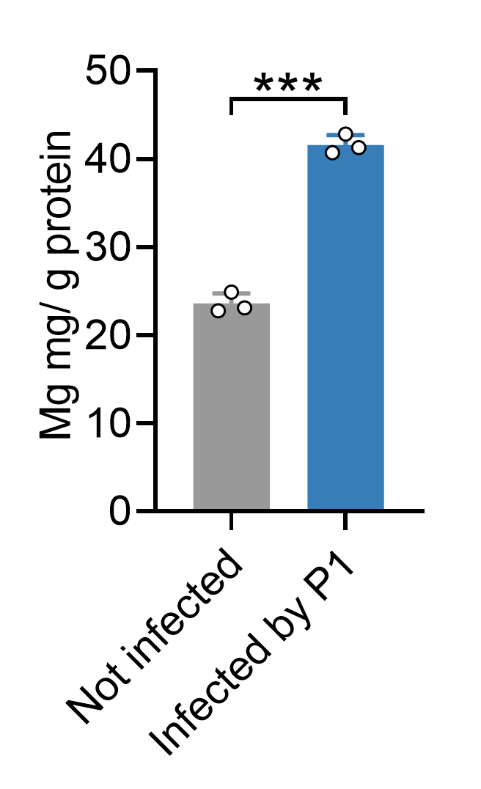


# Figure S1. After phage P1 infection, intracellular Mg^2+^ concentrations increase.

Phage P1 infected bacteria K12-MG1655 for 10 min (MOI = 0.1), the infected bacteria were centrifuged at 4500 rpm for 20 min to collect bacterial precipitation and washed with PBS buffer, and Mg^2+^ associated with bacterial cell was measured by ICP-MS. Uninfected bacteria were used as the control group. Data represent the mean ± SEM of three biological replicates, each of which was performed with three technical replicates. *P*  values were calculated using two-tailed Student’s *t*-test for paired comparisons. ****P* < 0.001.


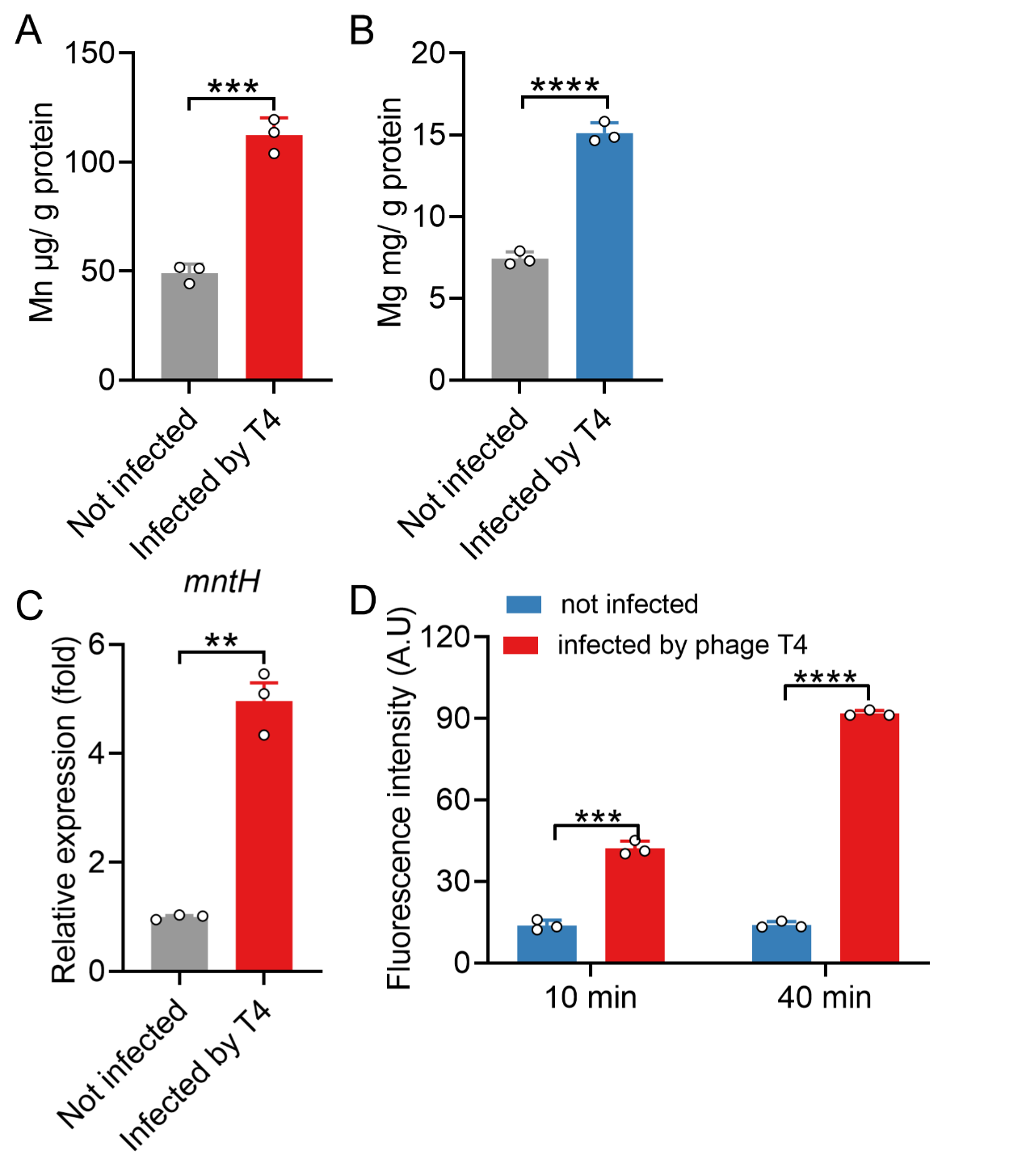


# Figure S2. The T4 phage infection increases intracellular Mn^2+^ concentrations and ROS level.

(A) and (B) T4 phage infected bacteria K12-MG1655 for 10 min (MOI = 0.1), the infected bacteria were centrifuged at 4500 rpm for 20 min to collect bacterial precipitation and washed with PBS buffer, and Mn^2+^ (A) and Mg^2+^ (B) associated with bacterial cell was measured by ICP-MS. Uninfected bacteria were used as the control group. (C) T4 infected K12-MG1655 for 10 min (MOI = 0.1), then the bacteria were collected and total RNA extracted. The expression of *mntH* was analyzed by qRT-PCR and normalized to *16S*. (D) T4 phage infected bacteria for 10 or 40 min (MOI = 0.1), the infected bacteria were centrifuged at 4500 rpm for 20 min to collect bacterial precipitation and washed with PBS buffer. The intracellular levels of ROS were determined with H_2_DCFDA dye. Fluorescence signals were measured using a SpectraMax M2 Plate Reader (Molecular Devices) with excitation/emission wavelengths of 488/525 nm. Data represent the mean ± SEM of three biological replicates, each of which was performed with three technical replicates. *P*  values were calculated using two-tailed Student’s *t*-test for paired comparisons or one-way analysis of variance (ANOVA) for multiple comparisons. ***P* < 0.01; ****P* < 0.001; *****P* < 0.0001.


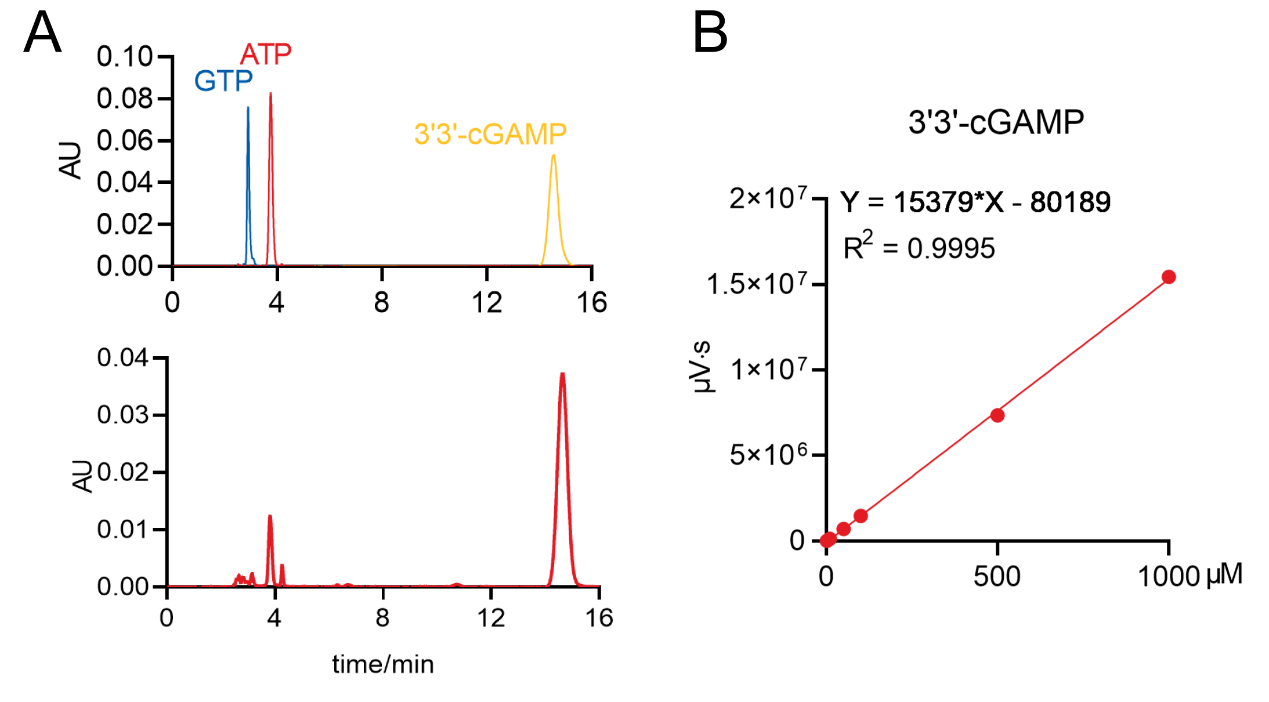


# Figure S3. HPLC for detecting the enzymatic activity of DncV.

(A) Top: the standard of ATP, GTP and 3'3'-cGAMP. Bottom: the enzyme-catalyzed reaction using ATP and GTP as substrates. 2 μM protein DncV was added into the reaction system (50 mM Tris-HCl, pH 7.5, 1 mM Mn^2+^, 100 μM ATP and 100 μM GTP) for 30 min at 37°C. Next, all reaction mixtures were heated for 10 min at 98°C and centrifuged for 10 min at 12000 rpm to obtain supernatant. Lastly, all samples were detected by HPLC. The wavelength of the UV detector was 254 nm. The supernatant was eluted with 98% Na_2_HPO_4_ (pH 5.2, 150 mM) and 2% acetonitrile in 16 min and the flow rate was 1 ml/min. (B) The standard curve of 3′3′-cGAMP standards. Different concentrations of 3′3′-cGAMP (1, 10, 100, 500 and 1000 μM) were measured by high-performance liquid chromatography (HPLC). A standard curve was generated based on the concentration of 3′3′-cGAMP.


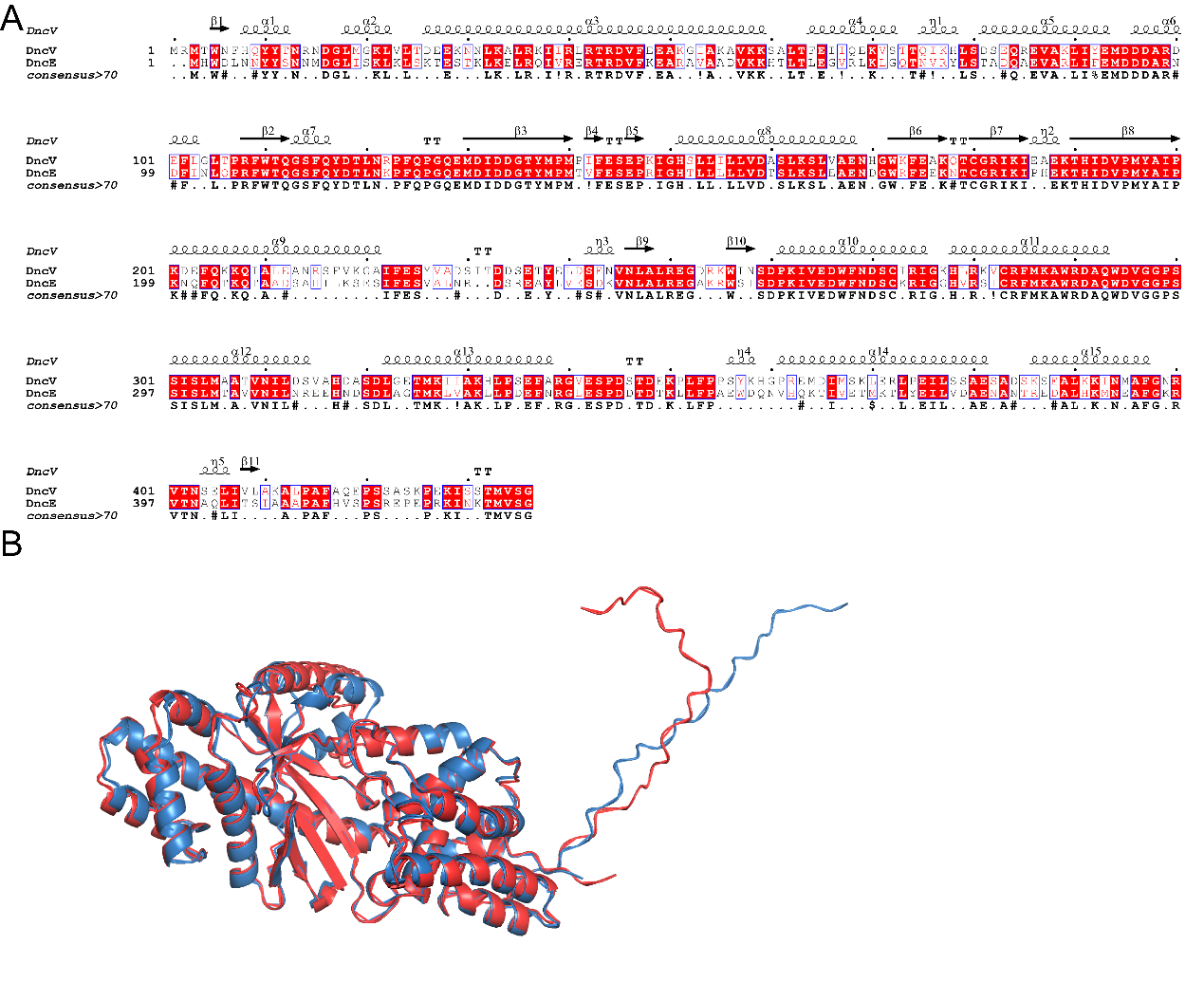


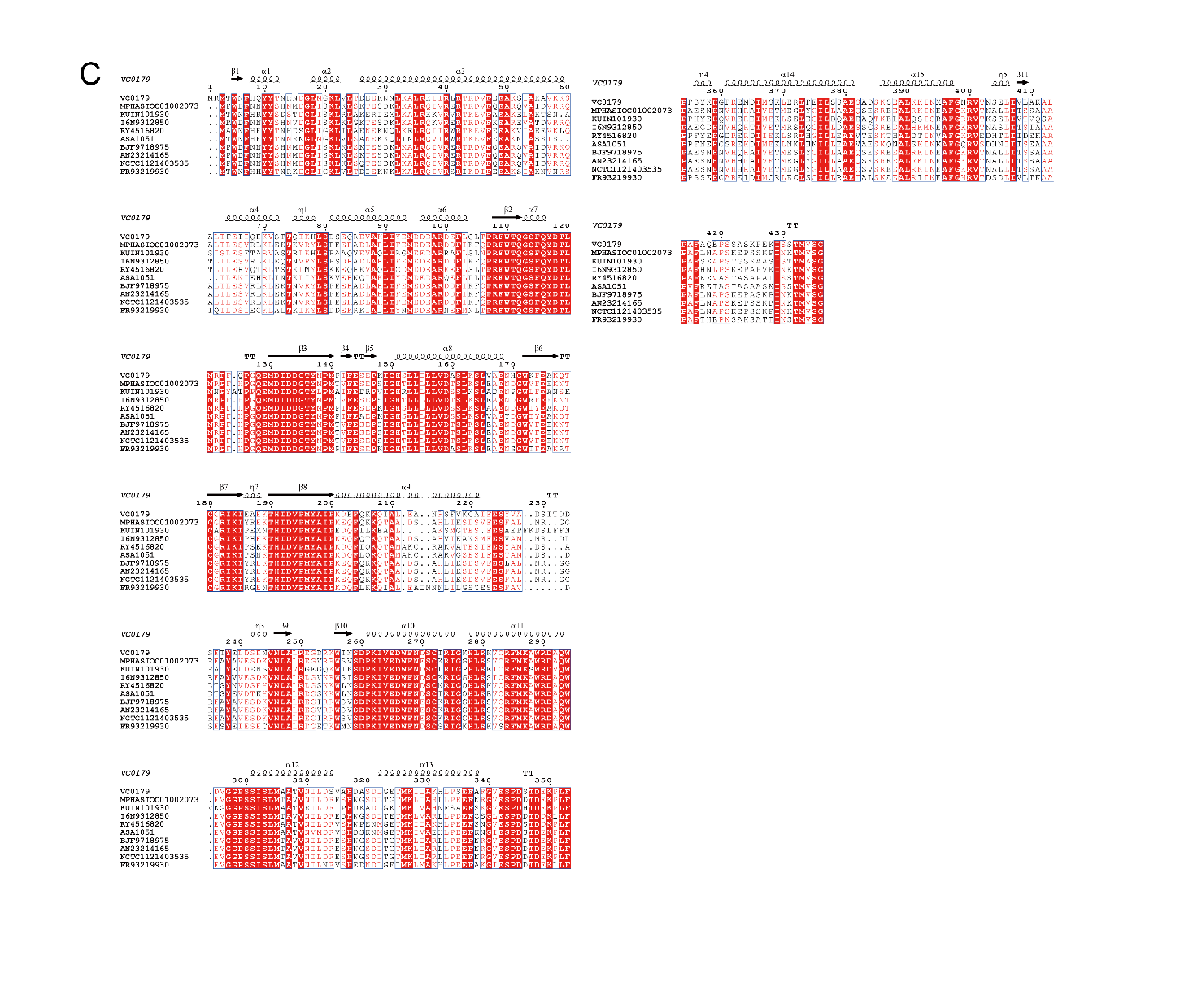


# Figure S4. The structure of DncV is highly similar to DncE.

(A) Alignment of the amino acid sequences of DncV and DncE. The amino acid sequences of DncV and DncE were acquired from NCBI. Protein sequences alignment was performed using DNAMAN software and visualized using ESPript3 software. (B) The 3D-structure of DncV and DncE proteins. Protein DncV and DncE structures were obtained from the PDB database. Structural visualization was made using PyMOL. The red represents the DncV protein, and the blue represents the DncE protein. (C) Protein sequence alignment of homologous DncV proteins. The protein DncV and the homolog protein sequences were obtained from NCBI databases. All protein sequences alignment was performed using DNAMAN software and visualized using ESPript3 software.


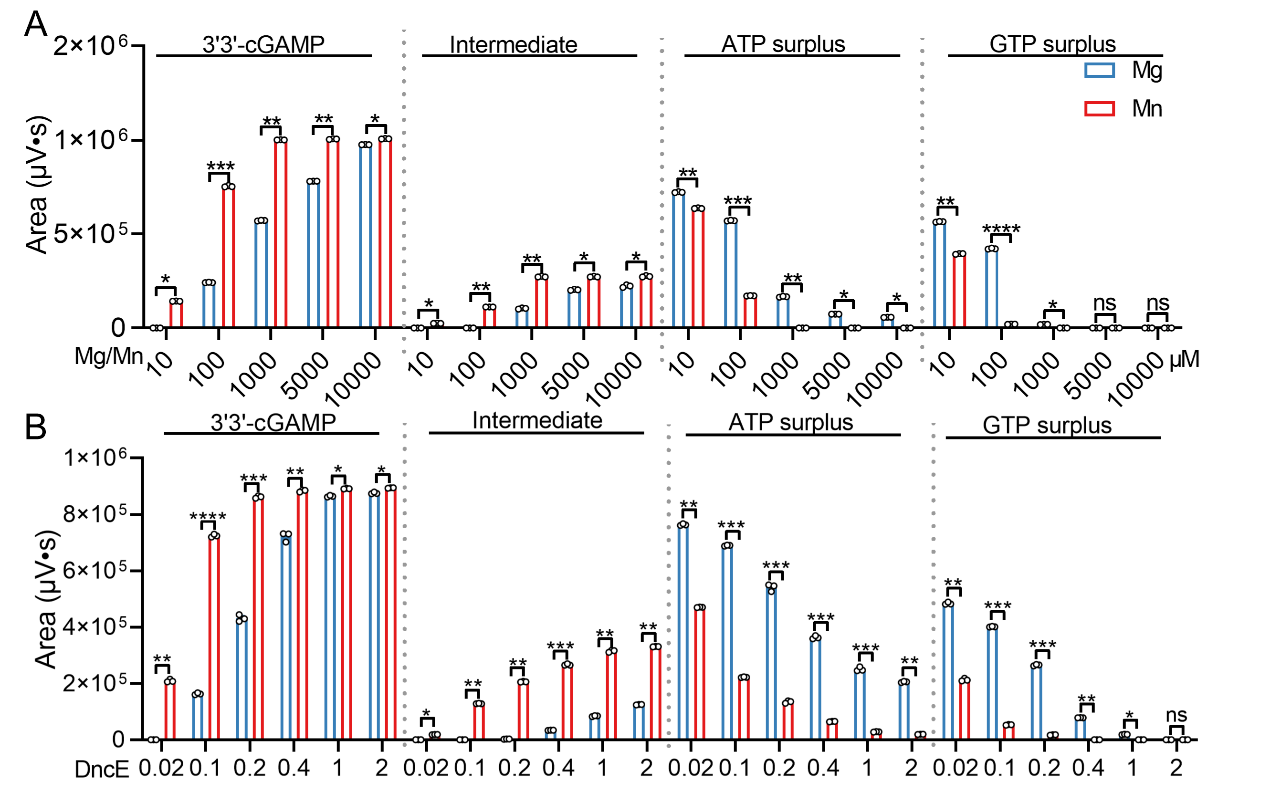


**Figure S5. Mn^2+^ boosts DncE's capacity to produce 3'3'-cGAMP..**

(A) Changing the Mg^2+^ or Mn^2+^ concentration (from 10 to 10000 μM), 2 μM protein DncE was added into the reaction system (50 mM Tris-HCl, pH 7.5, MgCl_2_ or MnCl_2_, 100 μM ATP and 100 μM GTP) for 30 min at 37°C. Next, all reaction mixtures were heated for 10 min at 98°C and centrifuged for 10 min at 12000 rpm to obtain supernatant. Lastly, all samples were detected by HPLC. The production levels of 3'3'-cGAMP and intermediate products, as well as the remaining level of ATP and GTP, in the Mg^2+^ or Mn^2+^-mediated reaction system were quantitatively analyzed using Empower software. (B) Changing the protein concentration (from 0.02 to 2 μM), different concentrations of the protein DncE was added into the reaction system (50 mM Tris-HCl, pH 7.5, 1 mM MgCl_2_ or MnCl_2_, 100 μM ATP and 100 μM GTP) for 30 min at 37°C. All other manipulations were as above. The production levels of 3'3'-cGAMP and intermediate products, as well as the remaining level of ATP and GTP, in the Mg^2+^ or Mn^2+^-mediated reaction system were quantitatively analyzed using Empower software.


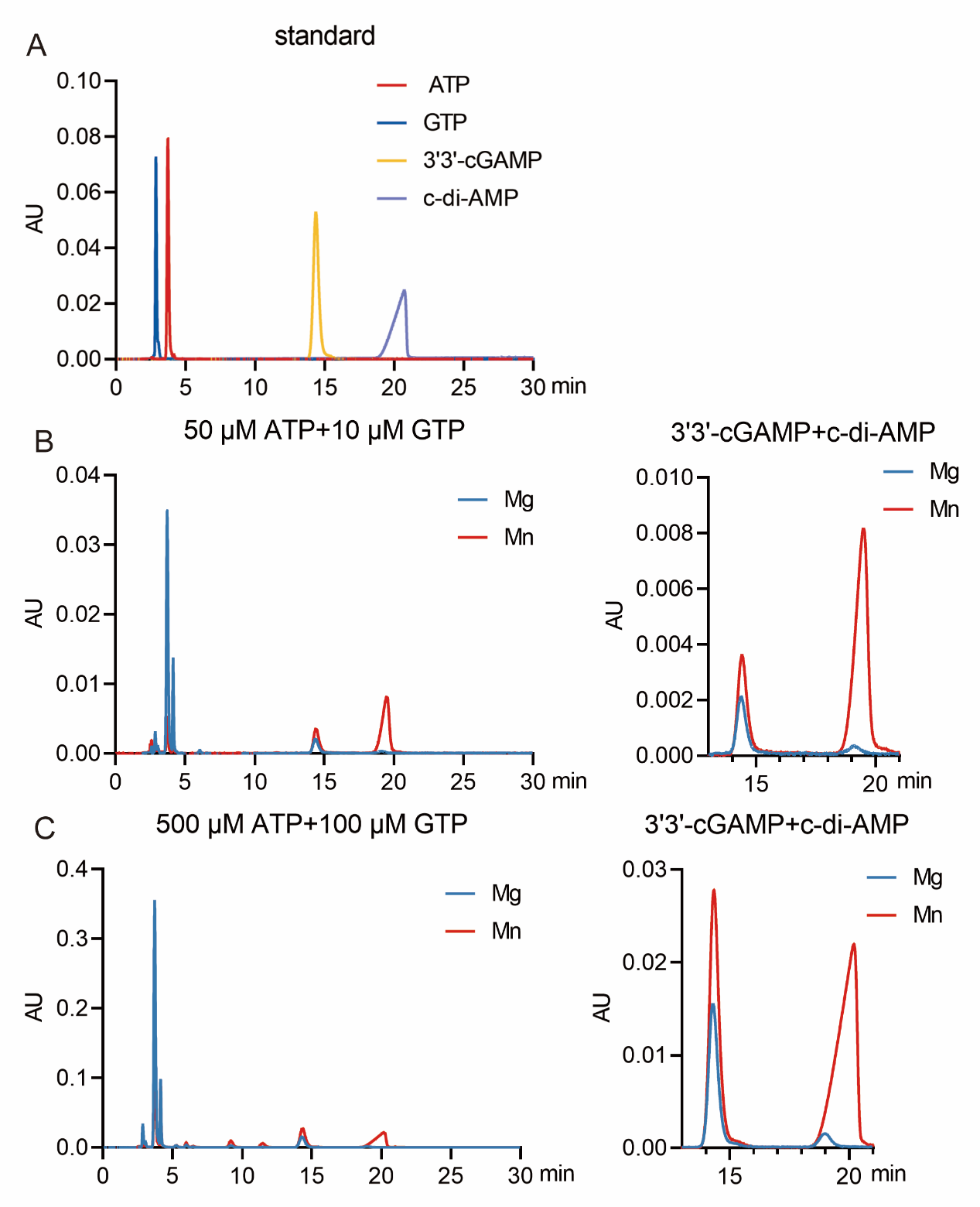


# Figure S6. Adding excessive ATP, the activation of DncV under Mn^2+^ reaction system was higher than Mg^2+^.

(A) Mixed HPLC chromatograms of ATP, GTP, 3'3'-cGAMP and c-di-AMP. (B) and (C) Changing the substrate concentration (5ATP/1GTP) (B: 50 μM ATP and 10 μM GTP, C: 500 μM ATP and 100 μM GTP), 2 μM protein DncE was added into the reaction system (50 mM Tris-HCl, pH 7.5, mM MgCl_2_ or MnCl_2_, ATP and GTP) for 30 min at 37°C. Next, all reaction mixtures were heated for 10 min at 98°C and centrifuged for 10 min at 12000 rpm to obtain supernatant. Lastly, all samples were detected by HPLC.


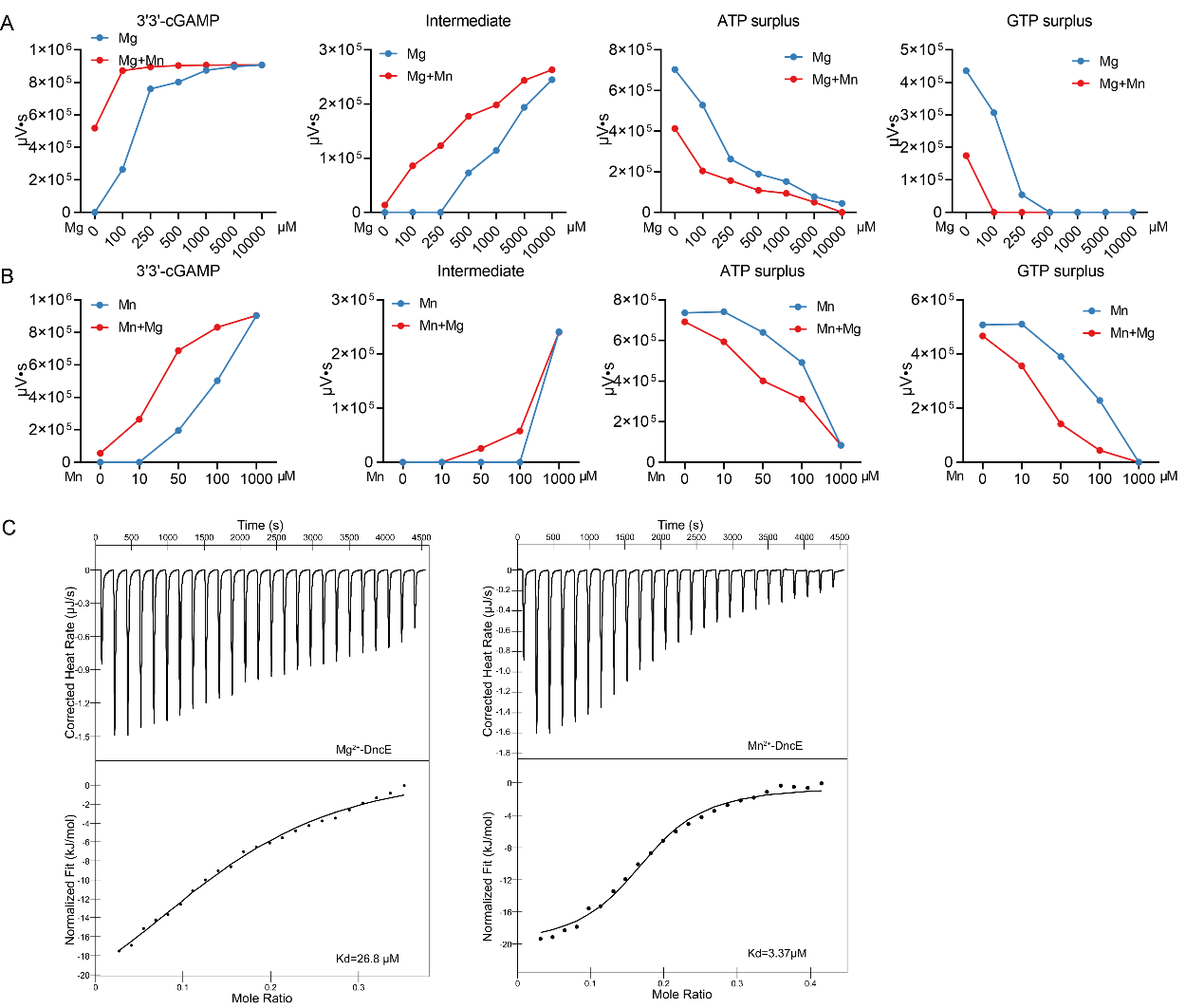


# Figure S7. Compared to Mg^2+^, Mn^2+^ can further enhance the enzymatic activity of DncE in Mg^2+^-mediated reactions.

(A) By adding the different concentrations of Mg^2+^ (from 0 to 10000 μM), without or with 10 μM Mn^2+^, 2 μM protein DncE was added into the reaction system (50 mM Tris-HCl, pH 7.5, MgCl_2_, 100 μM ATP and 100 μM GTP) for 30 min at 37°C. Next, all reaction mixtures were heated for 10 min at 98°C and centrifuged for 10 min at 12000 rpm to obtain supernatant. Lastly, all samples were detected by HPLC. The production levels of 3'3'-cGAMP and intermediate products, as well as the remaining level of ATP and GTP, were quantitatively analyzed using Empower software. (B) By adding the different concentrations of Mn^2+^ (from 0 to 1000 μM), without or with 250 μM Mg^2+^, 2 μM protein DncE was added into the reaction system for 30 min at 37°C. All other manipulations were as above. The production levels of 3'3'-cGAMP and intermediate products, as well as the remaining level of ATP and GTP, were quantitatively analyzed using Empower software. Data represent the mean ± SEM of three biological replicates, each of which was performed with three technical replicates. (E) The binding between Mg^2+^, or Mn^2+^ and DncE examined with ITC. Data were analyzed using the Nano Analyze software.


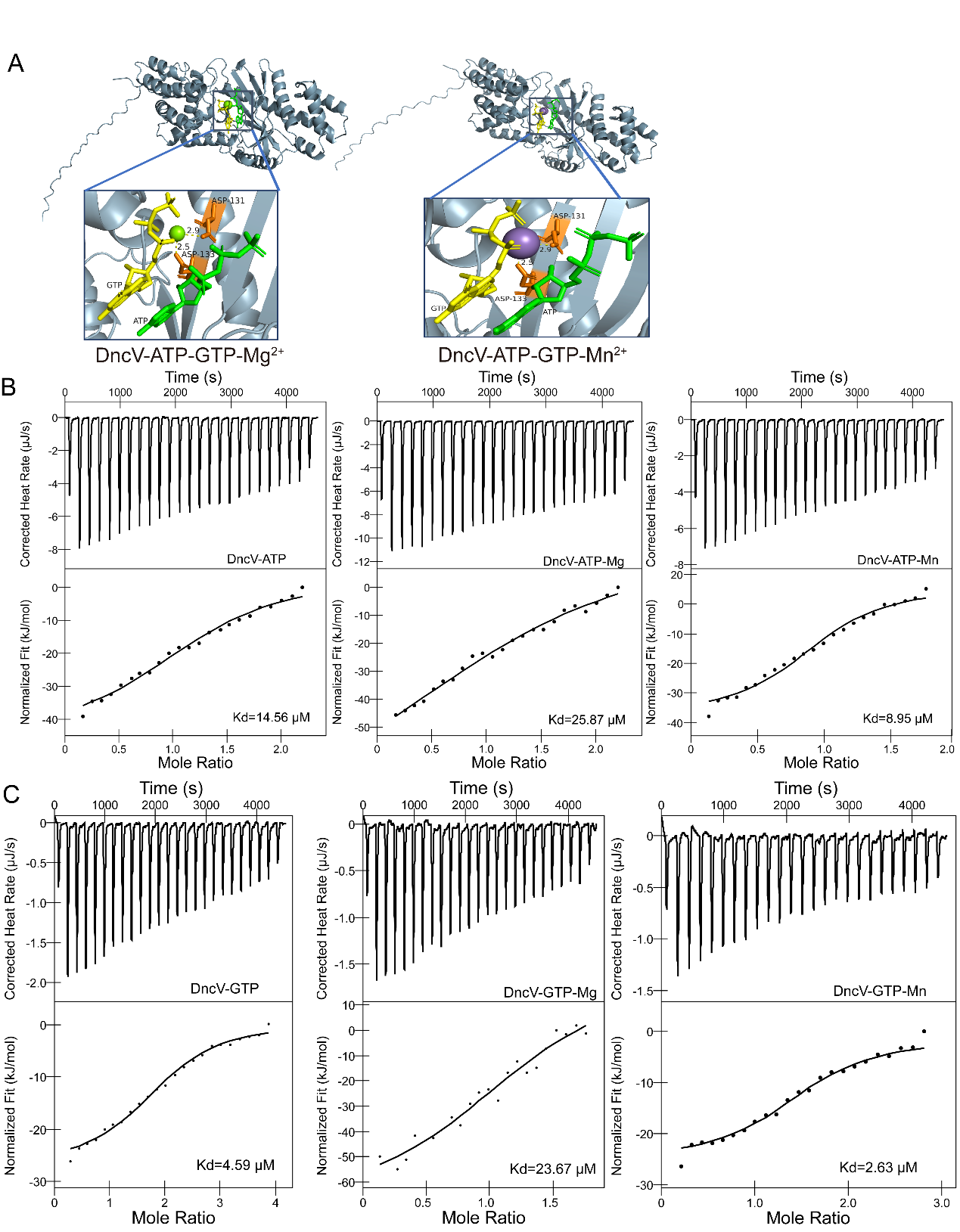


**Figure S8. Mn^2+^ can enhance the binding affinity of ligands for the DncV.**

(A) The structural model of Mg^2+^ and Mn^2+^ each docking with DncV-ATP-GTP, prepared using PyMOL. Mg^2+^ is shown as green sphere, Mn^2+^ is shown as purple sphere and ATP is shown in green and GTP is shown in yellow. (B) The binding of ATP to DncV, DncV-Mg^2+^or DncV-Mn^2+^ was examined by ITC. Data were analyzed using the Nano Analyze software. (C) The binding of GTP to DncV, DncV-Mg^2+^or DncV-Mn^2+^ was examined by ITC. Data were analyzed using the Nano Analyze software.


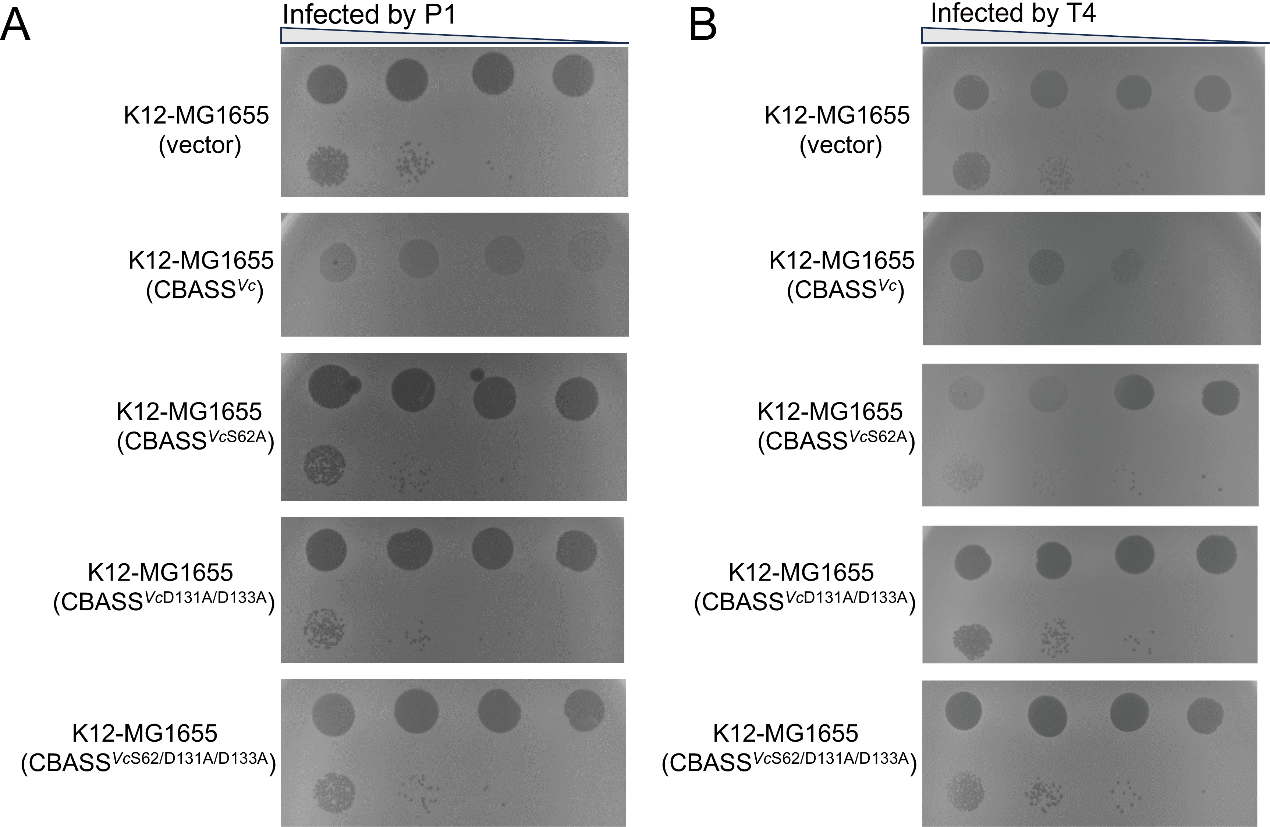


# Figure S9. The *Vibrio cholerae*-derived CBASS system conferred defense against phages.

(A) The P1 bacteriophage was serially diluted and plated onto different strains double-layer agar plates to test different strains resistance to the bacteriophage. (B) The T4 bacteriophage was serially diluted and plated onto different strains double-layer agar plates to test different strains resistance to the bacteriophage.


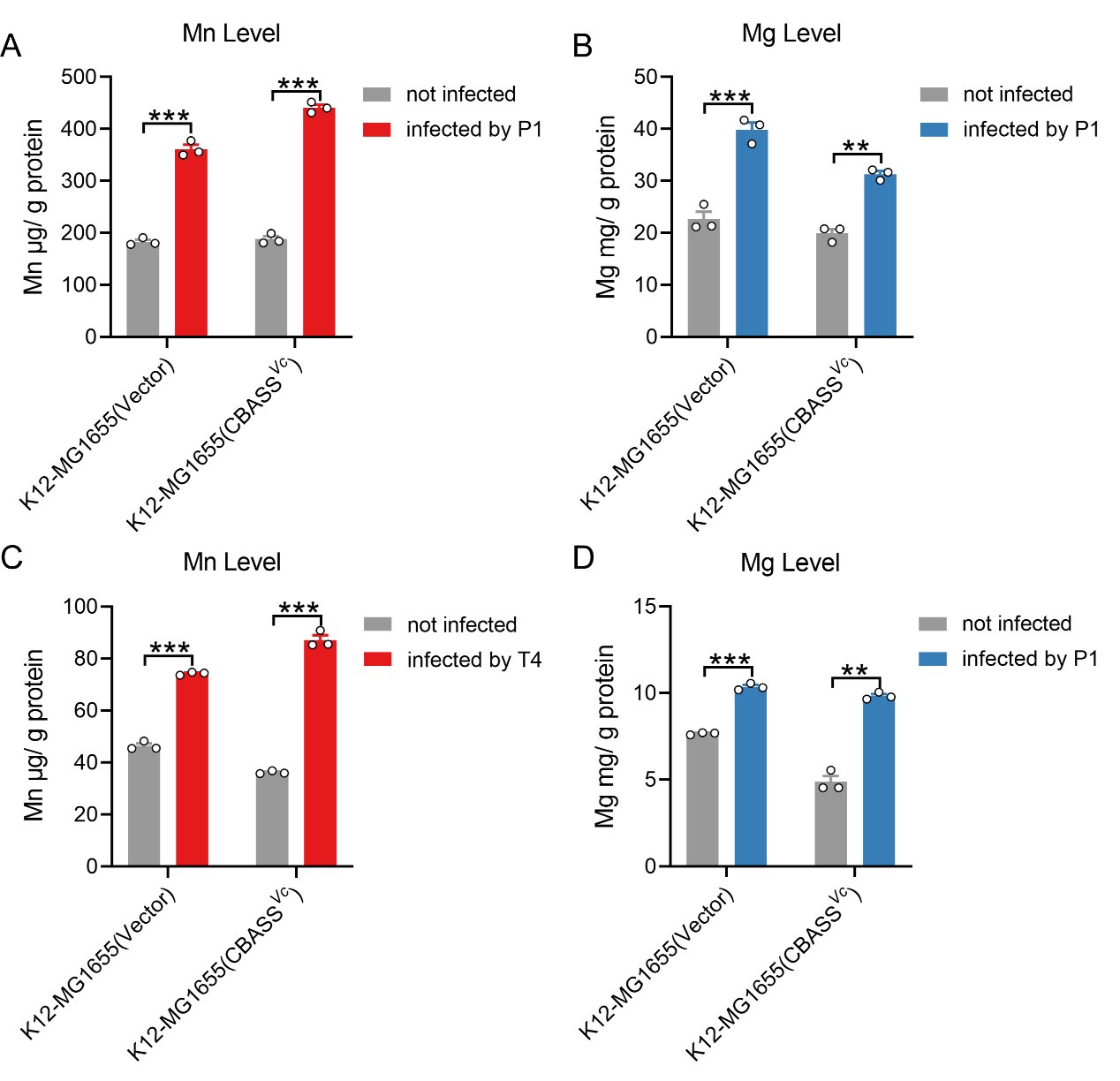


# Figure S10. After phage infection, intracellular Mn^2+^ concentrations increase.

(A) and (B) phage infected K12-MG1655 (vector) and K12-MG1655 (CBASS*^Vc^*) for 10 min (MOI = 0.1), the infected bacteria were centrifuged at 4500 rpm for 20 min to collect bacterial precipitation and washed with PBS buffer, and Mn^2+^ (A) or Mg^2+^ (B) associated with bacterial cell was measured by ICP-MS. (C) and (D) T4 phage infected K12-MG1655 (vector) and K12-MG1655 (CBASS*^Vc^*) for 10 min (MOI = 0.1), the infected bacteria were centrifuged at 4500 rpm for 20 min to collect bacterial precipitation and washed with PBS buffer, and Mn^2+^ (C) or Mg^2+^ (D) associated with bacterial cell was measured by ICP-MS. Data represent the mean ± SEM of three biological replicates, each of which was performed with three technical replicates. *P*  values were calculated using one-way analysis of variance (ANOVA) for multiple comparisons. ***P* < 0.01; ****P* < 0.001.


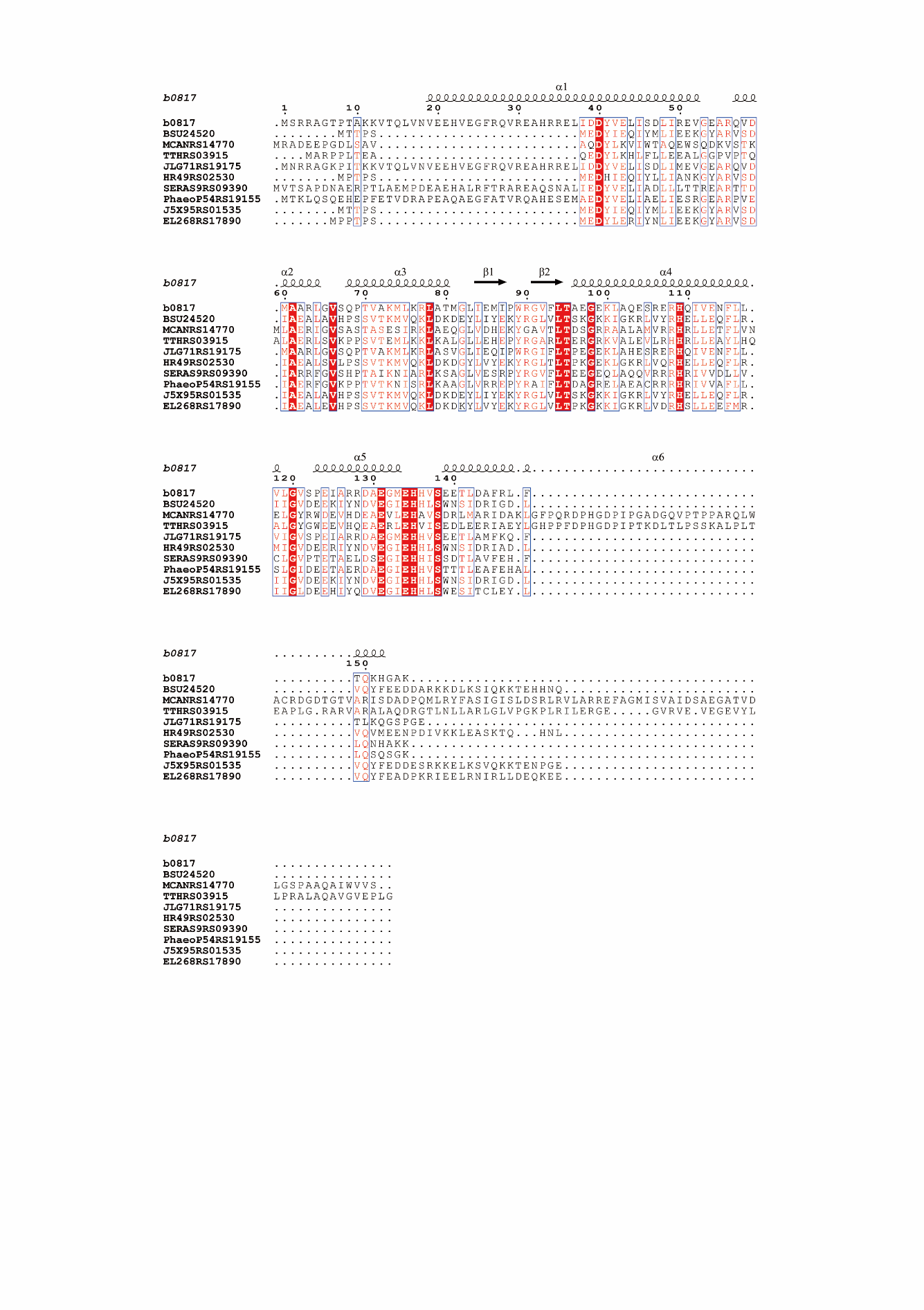


**Figure S11. The protein MntR is highly conserved in bacteria.**

Protein sequence alignment of homologous MntR (b0817) proteins. The protein MntR and the homolog protein sequences were obtained from NCBI databases. All protein sequences alignment was performed using DNAMAN software and visualized using ESPript3 software.


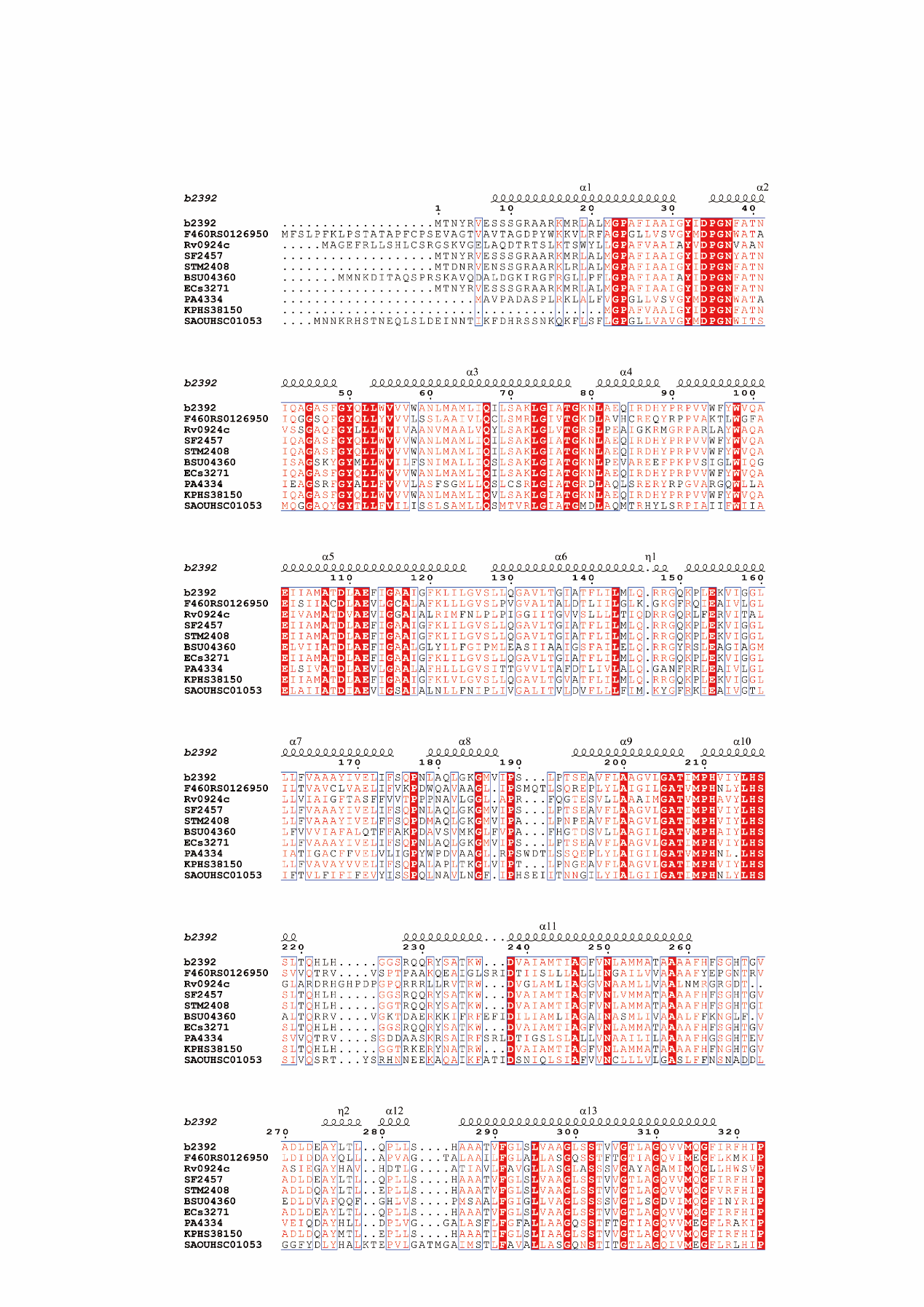


**Figure S12. The protein MntH is highly conserved in bacteria.**

Protein sequence alignment of homologous MntH (b2392) proteins. The protein MntH and the homolog protein sequences were obtained from NCBI databases. All protein sequences alignment was performed using DNAMAN software and visualized using ESPript3 software.


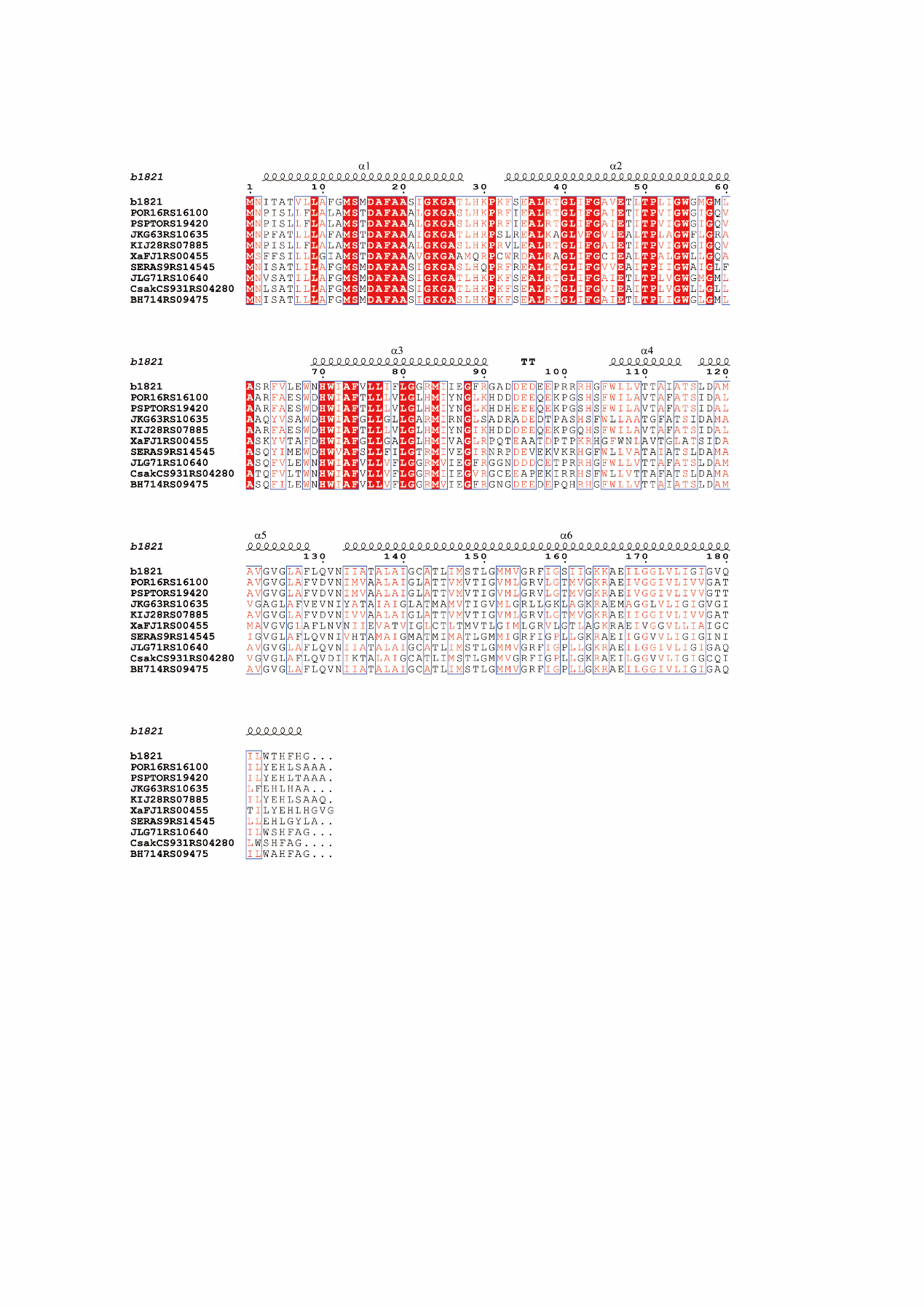


**Figure S13. The protein MntP is highly conserved in bacteria.**

Protein sequence alignment of homologous MntP (b1821) proteins. The protein MntP and the homolog protein sequences were obtained from NCBI databases. All protein sequences alignment was performed using DNAMAN software and visualized using ESPript3 software.

# Supplementary Tables

# Table S1. The bacterial strains used in the study.

| Strains | Relevant characteristics | References |
| --- | --- | --- |
| TG1 | *E. coli*, Host for cloning | Novagen |
| BL21 (DE3) | *E. coli*, Host for expression vector pET21a | Novagen |
| K12-MG1655 | *E. coli*, Wild-type strain, host for phage T4 & P1 | Dr. Yingfei Ma |
| K12-MG1655 (vector) | Wild-type strain containing pBBR1MCS-2, Km^r^ | This study |
| K12-MG1655 (CBASS*^Vc^*) | Wild-type strain containing pBBR1MCS-2-CBASS*^Vc^*, Km^r^ | This study |
| K12-MG1655 (CBASS*^Vc^*^D131A/D133A^) | Wild-type strain containing pBBR1MCS-2-CBASS*^Vc^*^D131A/D133A^, *cbass* sequence from *Vibrio cholerae*, Km^r^ | This study |
| K12-MG1655 (CBASS*^Vc^*^S62A^) | Wild-type strain containing pBBR1MCS-2-CBASS*^Vc^*^S62A^, *cbass* sequence from *Vibrio cholerae*, Km^r^ | This study |
| K12-MG1655 (CBASS*^Vc^*^S62A/D131A/D133A^) | Wild-type strain containing pBBR1MCS-2-CBASS*^Vc^*^S62A/D131A/D133A^, *cbass* sequence from *Vibrio cholerae*, Km^r^ | This study |
| Δ*mntH* | *mntH* gene deleted in K12-MG1655 | This study |
| Δ*mntH* (vector) | Δ*mntH* containing pBBR1MCS-2, Km^r^ | This study |
| Δ*mntH* (CBASS*^Vc^*) | Δ*mntH* containing pBBR1MCS-2- CBASS*^Vc^*, Km^r^ | This study |
| Δ*mntR* | *mntR* gene deleted in K12-MG1655 | This study |
| Δ*mntR* (vector) | Δ*mntR* containing pBBR1MCS-2, Km^r^ | This study |
| Δ*mntR* (CBASS*^Vc^*) | Δ*mntR* containing pBBR1MCS-2- CBASS*^Vc^*, Km^r^ | This study |
| Δ*mntP* | *mntP* gene deleted in K12-MG1655 | This study |
| Δ*mntP* (vector) | Δ*mntP* containing pBBR1MCS-2, Km^r^ | This study |
| Δ*mntP* (CBASS*^Vc^*) | Δ*mntP* containing pBBR1MCS-2- CBASS*^Vc^*, Km^r^ | This study |

# Table S2. The phages used in the study.

| Phages | Source | Identifier |
| --- | --- | --- |
| T4 | Dr. Yingfei Ma | N/A |
| P1 | ATCC 25404-B1 | N/A |

# Table S3. Plasmids used in this study.

| Plasmids | Relevant characteristics | References |
| --- | --- | --- |
| pBBR1MCS-2 | Broad-host-range vector, Km^r^ | Novagen |
| pBBR1MCS-2-CBASS*^Vc^* | CBASS*^Vc^* under the control of kanamycin resistance gene promoter in plasmid pBBR1MCS-2, Km^r^ | This study |
| pBBR1MCS-2-CBASS*^Vc^*^D131A/D133A^ | CBASS*^Vc^*^D131A/D133A^ under the control of kanamycin resistance gene promoter in plasmid pBBR1MCS-2, Km^r^ | This study |
| pBBR1MCS-2-CBASS*^Vc^*^S62A^ | CBASS*^Vc^*^S62A^ under the control of kanamycin resistance gene promoter in plasmid pBBR1MCS-2, Km^r^ | This study |
| pBBR1MCS-2-CBASS*^Vc^*^S62A/D131A/D133A^ | CBASS*^Vc^*^S62A/D131A/D133A^ under the control of kanamycin resistance gene promoter in plasmid pBBR1MCS-2, Km^r^ | This study |
| pCas | Crispr-Cas9 system plasmid used for in-frame deletion, Km^r^ | (1) |
| pTargetF1 | pTargetF with the spectinomycin resistance gene replaced by a chloramphenicol resistance gene, Cm^r^ | (2) |
| pTargetF1-Δ*mntH* | pTargetF1 derivative for *mntH* deletion in *E. coli* K12-MG1655 | This study |
| pTargetF1-Δ*mntR* | pTargetF1 derivative for *mntR* deletion in *E. coli* K12-MG1655 | This study |
| pTargetF1-Δ*mntP* | pTargetF1 derivative for *mntP* deletion in *E. coli* K12-MG1655 | This study |
| pET21a | Expression vector with N-terminal His tag, Amp^r^ | Novagen |
| pET21a-*dncV* | pET21a carrying *dncV* coding region, Amp^r^ | This study |
| pET21a-*dncV*^D131A/D133A^ | pET21a carrying *dncV*^D131A/D133A^ coding region, Amp^r^ | This study |
| pET21a-*dncE* | pET21a carrying *dncE* coding region, Amp^r^ | This study |
| pET21a-*capV* | pET21a carrying *capV* coding region, Amp^r^ | This study |
| pET21a-*capV*^S62A^ | pET21a carrying *capV*^S62A^ coding region, Amp^r^ | This study |

# Table S4. Primers used in this study.

| Primers | Sequence（5′ to 3′） | Function |
| --- | --- | --- |
| D-*mntH*-sg20-speI F | AGCTAGCTCAGTCCTAGGTATAATACTAGTGATCCCGAGTTTACCTACTTGTTTTAGAGCTAGAAATAGC | To generate pTargetF-sgRNA-Δ*mntH* |
| D-*mntH*-sg20 R | TTGCCGTTGTTGCGATTCTTTCAAAAAAAGCACCGACTCGG |  |
| D-*mntH*-up F | CCGAGTCGGTGCTTTTTTTGAAAGAATCGCAACAACGGCAA |  |
| D-*mntH*-up R | ATTCAACTACAATCCCAGCGCGCGATAGTTCGTCATCTTGTGC |  |
| D-*mntH*-down F | GCACAAGATGACGAACTATCGCGCGCTGGGATTGTAGTTGAAT |  |
| D-*mntH-*down SalI R | GGTAATAGATCTAAGCTTCTGCAGGTCGACCATCAATCAAATTACAAATTGGCG |  |
| D-*mntR*-sg20-speI F | AGCTAGCTCAGTCCTAGGTATAATACTAGTTTCTGACTTGATCAGGGAAGGTTTTAGAGCTAGAAATAGC | To generate pTargetF-sgRNA-Δ*mntR* |
| D-*mntR*-sg20 R | TTTGGTCTGAAAAACCCCACTTCAAAAAAAGCACCGACTCGG |  |
| D-*mntR*-up F | CCGAGTCGGTGCTTTTTTTGAAGTGGGGTTTTTCAGACCAAA |  |
| D-*mntR*-up R | GGCTCATTTGGCACCGTGTGCGCGACGACTCATTG |  |
| D-*mntR*-down F | CAATGAGTCGTCGCGCACACGGTGCCAAATGAGCC |  |
| D-*mntR*-down SalI R | GGTAATAGATCTAAGCTTCTGCAGGTCGACGCCAGCGCCTCAAAAATAAT |  |
| D-*mntP*-sg20-speI F | AGCTAGCTCAGTCCTAGGTATAATACTAGTTTTGTGCTGCTGATATTCCTGTTTTAGAGCTAGAAATAGC | To generate pTargetF-sgRNA-Δ*mntP* |
| D-*mntP*-sg20 R | GAGGTAATATAGCCTAAGCTATGTCTGATTCAAAAAAAGCACCGACTCGG |  |
| D-*mntP*-up F | CCGAGTCGGTGCTTTTTTTGAATCAGACATAGCTTAGGCTATATTACCTC |  |
| D-*mntP*-up R | CGTTCTTATTAACCGTGGAAGTGAGCAGTGATATTCATGACAATGTCC |  |
| D-*mntP*-down F | GGACATTGTCATGAATATCACTGCTCACTTCCACGGTTAATAAGAACG |  |
| D-*mntP*-down SalI R | GGTAATAGATCTAAGCTTCTGCAGGTCGACGTTTGCCGTTTTCCGATACC |  |
| *dncV* BamHI F | CGCGGATCCGTGAGAATGACTTGGAACTTTCACC | To generate pET21a-*dncV* |
| *dncV* XhoI R | CCGCTCGAGTCAGCCACTTACCATTGTGCTGCTG |  |
| *dncV*^D131A/D133A^ F | GTCAAGAAATGGCTATTGCTGATGGAACCTATATGCCAA | To generate pBBR1MCS-2-*cbass^D131A/D133A^* and pET21a-*dncV^D131A/D133A^* |
| *dncV*^D131A/D133A^ R | TTGGCATATAGGTTCCATCAGCAATAGCCATTTCTTGAC |  |
| *dncE* EcoRI F | CCGGAATTCATGCATTGGGATCTAAATAATTACTATAG | To generate pET21a-*dncE* |
| *dncE* XhoI R | CCGCTCGAGTTAGCCACTTACCATTGTTTTATTG |  |
| *capV* BamHI F | CGCGGATCCATGCCAAATCCACCTGAATATGA | To generate pET21a-*capV* |
| *capV* XhoI R | CCGCTCGAGTTACTTAAATTTGCGGGCAGGT |  |
| *capV*^S62A^ F | GACCTGATTACAGGTACTGCTATTGGTGGAATTCTGGCTTT | To generate pBBR1MCS-2-*cbass^S62A^*, pBBR1MCS-2-*cbass^S62A/D131A/D133A^* and pET21a-*capV^S62A^* |
| *capV*^S62A^ R | AAAGCCAGAATTCCACCAATAGCAGTACCTGTAATCAGGTC |  |
| RT K12-MG1655 *16S* F | GCAGGATGCGATGACCTTTA | For qRT-PCR |
| RT K12-MG1655 *16S* R | GTTATAAGGCAGGTTGCCGA |  |
| RT K12-MG1655 *mntH* F | GGATCGCGACTTTCCTGATT |  |
| RT K12-MG1655 *mntH* R | AACAACAGTAACCCGCCAAT |  |

Underlined indicate the restriction enzyme cutting sites.

# Table S5. Reagents and resources used in this study.

| Reagents or resources | Source | Identifier |
| --- | --- | --- |
| TRYPTONE | OXOID | #LP0042; CAS: 73049-73-7 |
| YEAST EXTRACT | OXOID | #LP0021; CAS: 8013-01-2 |
| NaCl | aladdin | #C111533; CAS: 7647-14-5 |
| MnCl_2_·(H_2_O)_4_ | aladdin | #M109463; CAS: 13446-34-9 |
| MnSO_4_ | aladdin | #M302973; CAS: 15244-36-7 |
| MgCl_2_ | aladdin | #M283904; CAS: 7786-30-3 |
| ZnCl_2_ | aladdin | #Z112532; CAS: 646-85-7 |
| FeCl_3_ | aladdin | #I112066; CAS: 7705-08-0 |
| CaCl_2_ | aladdin | #C290953; CAS: 10043-52-4 |
| CuCl_2_ | aladdin | #C433487; CAS: 7447-39-4 |
| ATP | Sigma-Aldrich | #A1852; CAS: 34369-07-8 |
| GTP | Sigma-Aldrich | #G8877; CAS: 36051-31-7 |
| c-di-AMP | Sigma-Aldrich | #SML1231; CAS: 54447-84-6 |
| c-di-GMP | Sigma-Aldrich | #SML1228; CAS: 61093-23-0 |
| 3′3′-cGAMP | Sigma-Aldrich | #SML1232; CAS: 849214-04-6 |

# REFERENCES

1. Jiang Y, Chen B, Duan C, Sun B, Yang J, Yang S. 2015. Multigene editing in the Escherichia coli genome via the CRISPR-Cas9 system. Appl Environ Microbiol 81:2506-14.

2. Zhang L, Li M, Li Q, Chen C, Qu M, Li M, Wang Y, Shen X. 2018. The Catabolite Repressor/Activator Cra Is a Bridge Connecting Carbon Metabolism and Host Colonization in the Plant Drought Resistance-Promoting Bacterium Pantoea alhagi LTYR-11Z. Appl Environ Microbiol 84.
